# Automated Coronary Optical Coherence Tomography Feature Extraction with Application to Three-Dimensional Reconstruction

**DOI:** 10.3390/tomography8030108

**Published:** 2022-05-17

**Authors:** Harry J. Carpenter, Mergen H. Ghayesh, Anthony C. Zander, Jiawen Li, Giuseppe Di Giovanni, Peter J. Psaltis

**Affiliations:** 1 School of Mechanical Engineering, University of Adelaide, Adelaide, SA 5005, Australia; anthony.zander@adelaide.edu.au; 2 School of Electrical Electronic Engineering, University of Adelaide, Adelaide, SA 5005, Australia; jiawen.li01@adelaide.edu.au; 3 Australian Research Council Centre of Excellence for Nanoscale BioPhotonics, The University of Adelaide, Adelaide, SA 5005, Australia; 4 Institute for Photonics and Advanced Sensing, University of Adelaide, Adelaide, SA 5005, Australia; 5 Vascular Research Centre, Lifelong Health Theme, South Australian Health and Medical Research Institute (SAHMRI), Adelaide, SA 5000, Australia; giuseppe.digiovanni@sahmri.com (G.D.G.); peter.psaltis@adelaide.edu.au (P.J.P.); 6 Adelaide Medical School, University of Adelaide, Adelaide, SA 5005, Australia; 7 Department of Cardiology, Central Adelaide Local Health Network, Adelaide, SA 5000, Australia

**Keywords:** atherosclerosis, biomechanics, border detection, coronary artery disease, optical coherence tomography, stents, vulnerable plaque

## Abstract

Coronary optical coherence tomography (OCT) is an intravascular, near-infrared light-based imaging modality capable of reaching axial resolutions of 10–20 µm. This resolution allows for accurate determination of high-risk plaque features, such as thin cap fibroatheroma; however, visualization of morphological features alone still provides unreliable positive predictive capability for plaque progression or future major adverse cardiovascular events (MACE). Biomechanical simulation could assist in this prediction, but this requires extracting morphological features from intravascular imaging to construct accurate three-dimensional (3D) simulations of patients’ arteries. Extracting these features is a laborious process, often carried out manually by trained experts. To address this challenge, numerous techniques have emerged to automate these processes while simultaneously overcoming difficulties associated with OCT imaging, such as its limited penetration depth. This systematic review summarizes advances in automated segmentation techniques from the past five years (2016–2021) with a focus on their application to the 3D reconstruction of vessels and their subsequent simulation. We discuss four categories based on the feature being processed, namely: coronary lumen; artery layers; plaque characteristics and subtypes; and stents. Areas for future innovation are also discussed as well as their potential for future translation.

## 1. Introduction

Coronary artery disease (CAD) is a leading cause of death, morbidity, and economic burden globally [1,2]. Although rates of myocardial infarction (MI) are decreasing through some parts of the world, recurrent major adverse cardiovascular events (MACE) following initial MI continue to occur at unacceptably high rates [3]. This is because of the complex pathogenesis and widespread nature of atherosclerotic plaques, including those in non-infarct related arteries that continue to pose a risk of plaque destabilization and atherothrombotic events [4,5]. This is despite advances in structural, molecular, and functional imaging technology, percutaneous coronary intervention (PCI) and pharmacotherapy. While invasive coronary angiography (ICA) is still the cornerstone of CAD assessment in real-world practice [6], intravascular imaging modalities, such as intravascular ultrasound (IVUS) and optical coherence tomography (OCT) can also be adjuvantly used, owing to their ability to identify vulnerable plaque features [7] such as plaque burden [8] and thin-cap fibroatheroma (TCFA) [9], respectively. These high-risk plaque features have been shown to portend up to a six-fold increase in future MACE [10]. However, the ability of conventional IVUS and OCT imaging to predict which plaques will progress to cause future thrombotic events is still suboptimal, with positive predictive values of only 20–30% [11].

Coronary biomechanics is emerging as a potentially useful tool to improve this predictive capability [12]. Computational fluid dynamics (CFD) has predominantly been applied to assess regions of low wall shear stress (WSS) [13,14,15], an established factor that has shown associations with low-density-lipoprotein deposition [16] and subsequent plaque progression [17,18]. Conversely, in the general population heightened structural stress [19,20] has been associated with plaque instability and rupture [21], as well as plaque growth over time [22], and can be modulated by the dynamics of left ventricular function [23,24,25]. This highlights the complex and highly nonlinear relationships within the coronary vasculature that can influence a patient’s biomechanical stress profile. Furthermore, the challenge facing coronary biomechanics, much like imaging modalities, is that no one parameter can provide a reliable or wholistic summation of a patient’s biomechanical profile. To address this, comprehensive biomechanical simulations are required, demanding high-fidelity imaging to segment important regions accurately and deliver robust, realistic, and patient-specific stress distributions.

**Figure 1:**
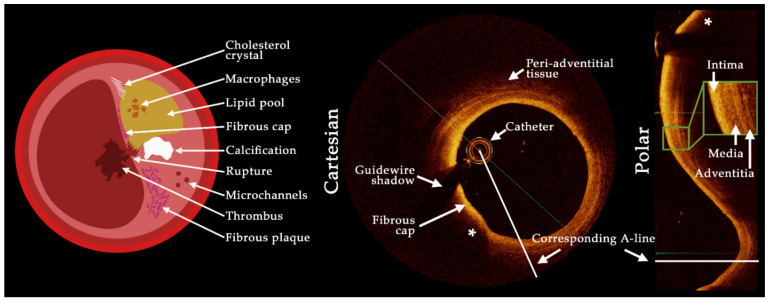
Schematic showing plaque features visible with optical coherence tomography (OCT) imaging as well as a visualization of A-lines in the cartesian and polar coordinates. The OCT images show a lipidic plaque (*) with fibrous cap and the delineation of the three artery wall layers is shown inset in the polar image representation. The limited penetration depth can be seen behind the lipidic component, with significant attenuation preventing visualization of the backside of plaque components.

Among current commercially available intracoronary imaging modalities applied in real-world clinical scenarios, OCT is uniquely placed to deliver sufficient accuracy, given that it has axial and lateral resolutions of 5–20 µm and 10–90 µm, respectively, depending on laser source and lens properties, approximately ten-fold higher axial and lateral resolutions than IVUS [26,27]. OCT achieves this accuracy through light-based, near-infrared spectrum wavelengths of 1250 to 1350 nm emitted from a single invasive fiberoptic wire, which rotates as it is pulled backwards through the target vessel [28]. The backscattering of light measured by the time for light to travel from tissue to the catheter lens over each revolution of the fiberoptic wire forms each cross-sectional image of the vessel wall. The high spatial resolution of this light-based imaging modality allows for delineation between atherosclerotic components [29,30], shown in Figure 1. This enables identification of high-risk features, notably thin fibrous cap, macrophage infiltration, plaque microchannels, cholesterol crystals, spotty calcification, lipid arc [31,32], and layering of plaque [33], which have been identified as predictors of rapid plaque growth [34] and determinants of biomechanical stress.

The primary limitation of commercially available intracoronary OCT is its penetration depth of 0.1 to 2 mm in plaques, compared to up to 10 mm for IVUS, which prevents visualization of the deep content of plaques, the external elastic membrane and adventitial layer in diseased regions [28,35]. This penetration depth decreases significantly in the presence of lipid rich plaques due to the high attenuation and low backscattering properties of lipid. However, OCT does overcome IVUS’s limited penetration depth in calcified lesions which ultrasound cannot penetrate. Despite this, many clinical studies have taken OCT-centered approaches [36,37,38,39] to assess vulnerable plaque features or biomechanically simulate arteries after three-dimensional (3D) reconstruction [40,41,42,43,44]. Nevertheless, annotation of OCT images is still predominantly a manual and tedious task, susceptible to individual interpretation, which is a major obstacle to its use [45]. Indeed, the risk of intra and inter-observer variability in quantitative analysis necessitates that each image is analyzed by at least two analysts, further compounding the significant time cost.

**Figure 2:**
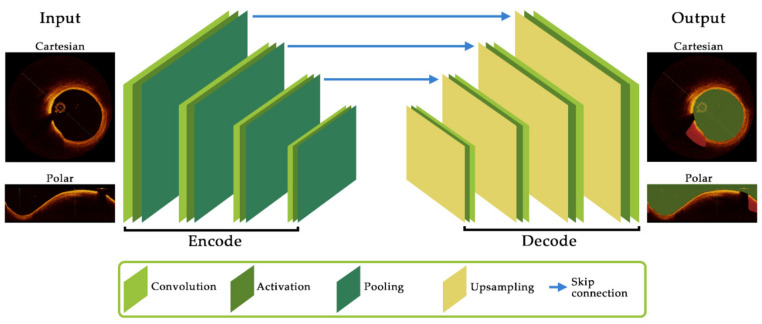
Schematic of key components and their layout for a convolutional neural network architecture. The encoder component consists of convolution and activation functions to extract feature maps before pooling (downsampling) to the subsequent layer. The decoder up-samples feature map data before further convolutions. Skip connections allow feature map data to be passed between layers which can assist in reducing resolution degradation between layers and is a critical feature of the popular U-Net architecture.

With the advent of machine learning techniques, automated medical image classification and segmentation has gained significant attention, with deep learning based neural networks predominantly used for medical image analysis [46]. In the simplest terms, these models work through back-propagation to minimize a prescribed loss function (such as cross-entropy [47], dice loss [48] or Tversky [49]) by directing a machine how to alter its parameters. The most common method used in image analysis is a convolutional neural network (CNN) [50]. Compared to artificial neural networks (ANNs) [51], that work by connecting multiple inputs to individual neurons, which are then multiplied by a weight and effectively summed to produce a single output, CNNs can reduce the number of weights used through sharing, resulting in convolution operations, and reduced computation time. CNNs generally apply a combination of convolutional and pooling layers, where the pooling layer down samples data allowing for an increased field of view in subsequent layers, as described in Figure 2. However, this usually leads to a reduction in image resolution [52], which can hamper the accurate segmentation of tissue borders, a critical feature for biomechanical simulation. Fully convolutional networks (FCN), such as the U-Net [53] which is named after its characteristic U-shaped structure, can assist in meeting this challenge. These networks couple the high-resolution, low level image data with low-resolution, higher level feature information to improve image segmentation and classification results. Various architectures exist depending on the task to be completed and interested readers are directed to references [54,55,56,57,58] for more detail.

**Figure 3:**
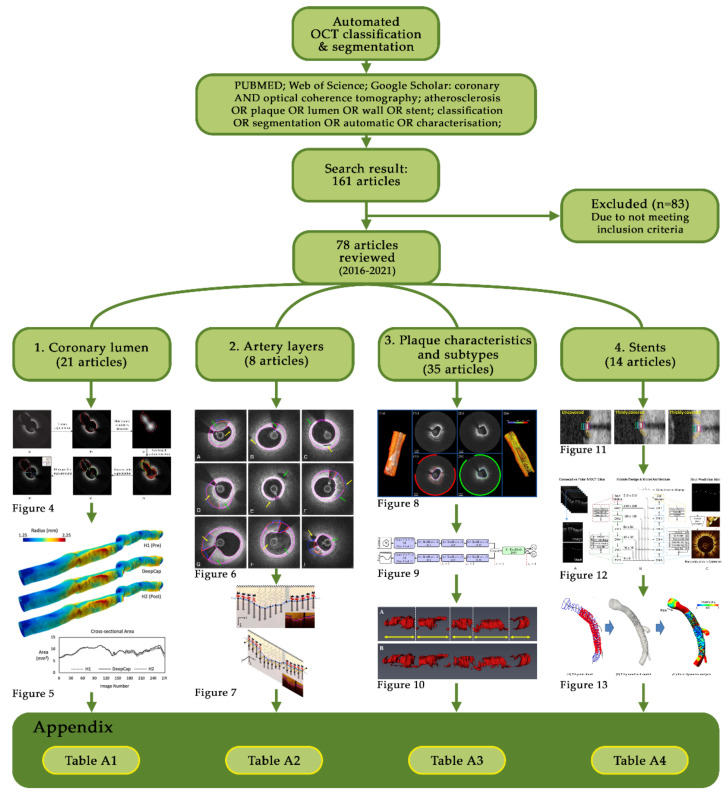
Consort diagram showing the review layout and Appendix A tables for each section.

In this systematic review, we evaluate recent methods to automatically segment and classify pathological and non-pathological features in coronary OCT imaging. This automated segmentation is critical to rapidly and quantitatively assessing atherosclerotic lesions in clinical scenarios. Uniquely, we focus this review on the application of automated techniques to 3D computational reconstruction and subsequent patient-specific simulation which requires specific characteristics to be accurately delineated, such as the outer elastic membrane and deep plaque components. PUBMED and Web of Science databases were searched, supplemented by Google Scholar, resulting in 161 articles which were further screened based on title and abstract to include only full-length, original journal articles published during the previous five years (2016–2021). Figure 3 details the consort diagram and review categories. A total of 78 screened articles were classified based on their focus as either the coronary lumen, artery layers, plaque characteristics and subtypes and stents. Included articles are summarized in Appendix A (Table A1, Table A2, Table A3 and Table A4), classifying the aim, dataset size, morphological/filter operations, feature detection/classification method, presented outcome and the point of comparison of each study. A glossary of evaluation metrics used to assess algorithm performance is also provided. Finally, we highlight potential challenges and multi-disciplinary opportunities for the computer science, engineering, and medical fields.

## 2. Coronary Lumen

Segmentation of the coronary artery lumen contour is perhaps the simplest task for automated techniques when there is no atherosclerotic disease and there has been appropriate clearance of blood from the OCT images. Here, globally used binarization methods [59], such as Otsu filtering [60,61,62,63], morphological operations, edge detection [64,65,66] and curve fitting [67] were often sufficient to automatically delineate the lumen. However, these methods are challenged when facing bifurcation regions and catheter artefacts, as well as improper blood clearance, which are not uncommon occurrences in clinical scenarios. Using a sequential combination of processing steps, an automated lumen border detection tool has shown good agreement with expert annotation when addressing these challenges [63]. Tissue characteristics, such as reflectivity, backscattering and absorption were used followed by contour refinement with a weighted linear least squares local regression approach before fitting of a second-degree polynomial to bridge catheter and bifurcation artefacts. However, these approaches can suffer in more complex lumen geometries, difficult bifurcation contours and stented artery sections.

Addressing complex lumen geometries, Joseph et al., developed a lumen segmentation method by enhancing lumen intensity through a transmittance-based method to iteratively drive the detected lumen edge towards the true lumen contour [68]. By utilizing speckle properties through a localized level-set segmentation method, this approach showed the ability to overcome image intensity variations. This allowed segmentation of challenging imaging datasets, including multiple lumens and subsequent automated 3D reconstruction. Other approaches to difficult lumen geometries include random walks based on edge weights and optical backscattering and graph-cut segmentation [69,70].

The latter, investigated by Essa et al., introduced a spatio-temporal segmentation method applying a Kalman filter to ensure border homogeneity and smoothness across an entire pullback [70]. This assisted in overcoming localized image-based noise and artefacts, an important consideration in automated 3D reconstruction. A cost function based on asymmetric local phase and first-order gaussian derivatives was introduced alongside a set of shape constraints to train a random forest (RF) classifier [71]. RF is particularly useful when handling noisy data and a large amount of input features as it avoids over fitting and can be more computationally efficient than other supervised learning techniques such as support vector machines (SVM) [72]. This approach achieved a sensitivity, specificity and Jaccard similarity index of 95.55 ± 3.19%, 99.84 ± 0.29%, and 0.95 ± 0.03, respectively, improving upon earlier first-order gaussian derivative approaches that achieved 89.76 ± 5.99%, 99.80 ± 0.56%, and 0.89 ± 0.06 in the same metrics [73]. Compared to using image intensity values alone, classification accuracy increased 6.80% in a dataset of 1846 images from 13 pullbacks (457 training, 1389 testing), whilst the mean average difference in area and the Hausdorff distance were reduced by 55% and 70% respectively. This highlights both that evaluation metric heterogeneity can significantly bias how improvement is measured, and that spatio-temporal approaches that consider all images in a pullback can achieve smooth contour segmentation in complex lumen geometries.

Although it is common to ignore bifurcation regions in 3D reconstructions, these regions are important to consider when assessing hemodynamics due to their flow-disturbing nature. However, bifurcation regions present difficulties when automatically segmenting the lumen. Addressing this, Macedo et al., built on their earlier work to propose a distance transform, similar to the distance regularized level set proposed in [74], to automatically correct lumen segmentation in bifurcation regions and areas of complex plaque [62,75]. Regions of bifurcations achieved results of 1.20 ± 0.80 mm^2^ and 0.88 ± 0.08 for the mean average difference in area (MADA) and dice coefficient, respectively, compared to manual segmentation. This was in comparison to non-bifurcation regions achieving 0.19 ± 0.13 mm^2^ and 0.97 ± 0.02 in the same metrics. Rather than a distance transform, Akbar et al., proposed an L- and C-mode interpolation approach to bridging lumen contour gaps caused by bifurcations [65]. Their approach, applied to 5931 images (40 patients), was then used to automatically reconstruct 3D lumen models for fractional flow reserve (FFR) assessment, with good correlation between manual and automated segmentations (R = 0.98).

**Figure 4:**
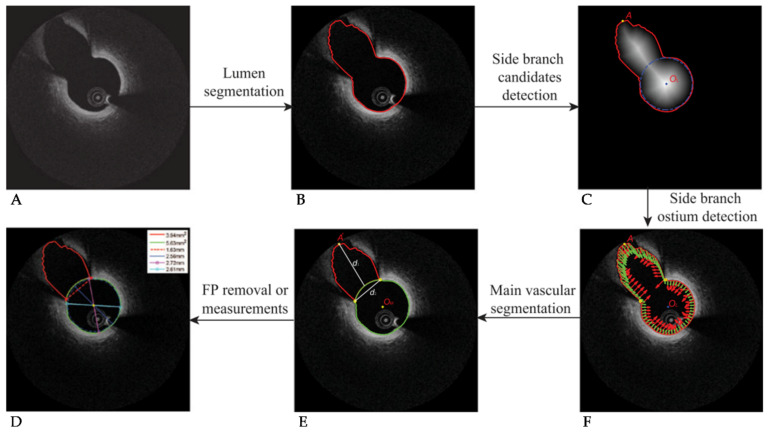
Visualization of the bifurcation identification method. (**A**) Original OCT image with bifurcation present. (**B**) Contour detection around lumen and branch. (**C**) Distance transform and the determined main vessel and side vessel centroids. (**D**) Final segmented image. (**E**) Detection of the side branch ostium location. (**F**) Normal vectors to the contour surface (red) and vectors pointing to the main vessel center (green). © [2017] IEEE. Reprinted, with permission, from [76].

To automatically segment bifurcation regions, rather than simply bridging over them, Cao et al., developed an automated branch ostium detection method [76]. By first fitting a contour to the main lumen, a dynamic programming based distance transform, introduced earlier and visualized in Figure 4c [74], was then used to select the main lumen and branch centroids. Ostium points on the main lumen contour were then detected using a differential filter and taking locations of maximum curvature. The method, shown in Figure 4, resulted in reasonable agreement to manual segmentation, but required manual intervention to adjust the threshold for the elliptical ratio of branches to avoid misclassification. Further advancement of this method by using a bifurcation classifier, such as that proposed by Miyagawa et al., could enhance segmentation results [77]. By comparing four CNNs (an original network using stochastic gradient descent followed by three networks making use of transfer learning from previous investigations [78]) a final area under the curve (AUC) of 99.72 ± 0.17% was reached, outperforming other bifurcation classifiers [75,79,80]. Interestingly, no statistically significant difference was found between results using polar and cartesian image coordinates, removing the need to pre-process images to polar form.

To improve the ability to classify and segment the lumen in difficult regions, such as stented arteries and bifurcations, machine learning approaches show significant potential. Yang et al., compared the performance of six classifiers (RF, SVM, J48, Bagging, Naïve Bayes and adaptive boosting (AdaBoost) [81,82,83]) in difficult or irregular regions [84]. By identifying and classifying 92 features from 54 patients and 14,207 images (1857 images denoted as irregular) through supervised learning and a partition-membership filtering method, the RF classifier produced the best overall accuracy compared to the other five classifiers: RF 98.2%, SVM 98.1%, J48 97.3%, Bagging 96.6%, Naïve Bayes 88.8%, AdaBoost 88.7%. However, residual blood artefacts and clots hampered accuracy, which Yong et al., subsequently improved upon with a linear regression CNN trained on a 64 pullback dataset (19,027 images) [85]. Consisting of four convolutional layers and three fully connected layers with gradient based adaptive optimization (ADAM) [86], an overall dice and Jaccard index of 0.99 and 0.97 were reached, respectively, with an average processing time of 40.6 ms per image. Here the most significant improvements in accuracy were seen after training on 25 pullbacks; however, incremental gains were seen by including additional images.

As networks deepen, detailed information can be gradually lost due to resolution degradation, hampering classification and segmentation accuracy. Tang et al., addressed this by proposing a novel N-Net based CNN capable of re-using the original input image in deeper convolutions to couple the initial high resolution data with low resolution feature information [87]. Consisting of a multi-scale U-Net architecture and cross-entropy loss function trained on 20,000 images, results showed excellent agreement to expert annotation, including in complex lumen shapes, such as bifurcation regions (accuracy: 0.98 ± 0.00; specificity: 99.40 ± 0.05%; dice: 0.93 ± 0.00). The N-Net also resulted in significantly reduced loss (0.08) compared to traditional U-Net architectures (0.11–0.15). Approaches like this could assist in accurately and efficiently generating 3D lumen geometries for assessment of quantitative flow reserve (QFR) or WSS in near-real time [88,89,90].

**Figure 5:**
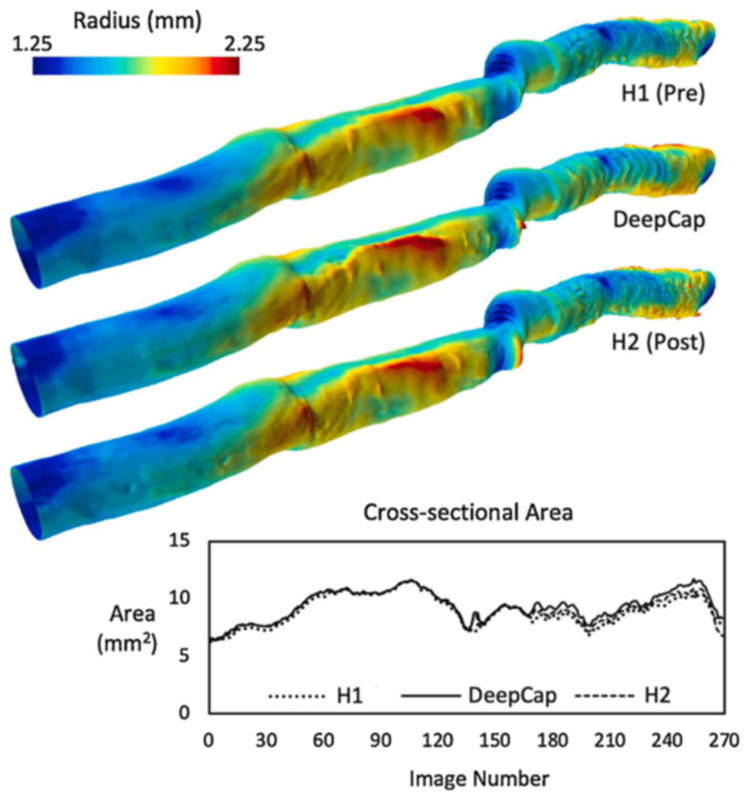
A comparison between the proposed DeepCap model and two manually annotated reconstructions (H1 and H2). The proposed model agrees well with both manual reconstructions, with the 3D lumen surface visualizing the radius measured from the lumen centroids and the graph showing the cross-sectional area along the length of the vessel. The automated DeepCap segmentation was able to process the 200-image pullback in just 0.8 s on a GPU (19 s on CPU). Reprinted from [93], with permission from Elsevier.

For clinical application, computationally efficient segmentation and simulation is important. Using the K-means algorithm for unsupervised learning, followed by B-spline curve fitting, Athanasiou et al., achieved significant computation speed-ups compared to their previous methods [91,92]. A total computation time of 180 sec for lumen border detection and 3D reconstruction was achieved using biplane angiography. This compared to 1080 sec previously, with added robustness in cases with artefacts and noise, resulting in excellent agreement between manual and automated WSS computations (R^2^ = 0.95). Computational speed and efficiency were further improved during the development of DeepCap, which further focused on using a small memory footprint [93]. Their approach was based on a U-Net architecture, using upsampling, downsampling and skip connections to improve network gradient propagation [94]. Dynamic routing was then utilized to optimize capsule weights [95,96]. Comparisons made between the UNet-ResNet18 (UNet-18), FCNResNet50 (FCN-50) and DeepLabV3-ResNet50 (DLV3-50) [97,98,99] showed that the proposed DeepCap method achieved 70% faster graphics processing unit (GPU) computation, 95% faster central processing unit (CPU) computation and a 70% reduction in memory. This speedup resulted in segmentation of an entire 200 image pullback in 19 sec on a CPU and just 0.8 sec on a GPU. This was achieved with comparable robustness and accuracy (dice: 97.00 ± 5.82; Hausdorff distance: 3.30 ± 1.51; specificity: 99.54 ± 0.75%; sensitivity: 93.27 ± 8.22%) in a 12,011 image (22 patient) dataset. Impressively, only 12% of the total parameters of previous methods were used. The resulting 3D reconstruction and comparison to expert annotation-based reconstructions is shown in Figure 5. This rapid clinical application of automated lumen segmentation could produce a significant leap in quantitative data available to clinicians, improving patient outcomes and the utility and acceptance of intravascular imaging modalities, machine learning approaches and the translation of 3D simulation capability, such as WSS computation.

## 3. Artery Layers

In healthy coronary sections the inner and outer elastic membranes can be visualized through intensity changes and their associated gradients, as illustrated previously in Figure 1. Using this knowledge, Zahnd et al., developed a front propagation scheme to segment the intima-media, media-adventitia and adventitia-periadventitial tissue borders [100]. By using the image gradient properties, an AdaBoost classified machine learning approach, and feature selection based on a RF framework, segmentation errors of 29 ± 46 µm, 30 ± 50 µm and 50 ± 64 µm resulted for the intima-media, media-adventitia and adventitia-periadventitial layers (Dice = 0.93). By further investigating the efficacy of three emerging classifiers (CNN pre-trained on the AlexNet model, RF and SVM), Abdolmanafi et al., found that the most robust feature extractor was the pre-trained CNN, while the RF produces the best classification results of up to 96% for the media layer [101]. Furthermore, using the pre-trained CNN as a feature generator for both the RF and SVM classifiers resulted in their highest accuracy (96 ± 0.06 and 0.90 ± 0.10, respectively) and most computationally efficient approach compared to the purely CNN method (0.97 ± 0.04).

Further approaches to segment the intimal and medial layers in cardiac allograft patients made use of the layered optimal graph-based image segmentation for multiple objects and surfaces (LOGISMOS) framework [73,102,103,104,105]. This approach enables a fast and quantitative assessment of changes in wall morphology that associate with cardiac allograft vasculopathy (CAV). By using transfer learning from the ImageNet database initialized with the Caffe framework [106], Chen et al., generated exclusion regions to classify artery layers in 50 heart transplant patients, with average errors of 4.98 ± 31.24 µm and 5.38 ± 28.54 µm for the intima and media respectively [102]. These errors were less than the inter-observer variability reported of 6.76 ± 10.61 µm, although their standard deviations were significantly larger, possibly due to the surface smoothness constraint put on the algorithm.

**Figure 6:**
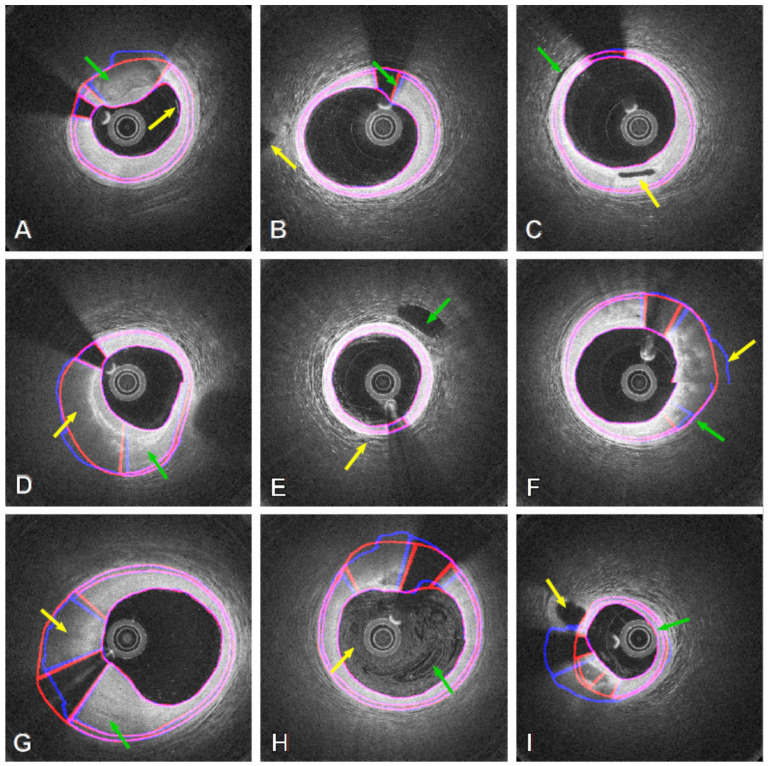
Results obtained from both the automatic method (blue contours) and expert annotation (red contours) in PS-OCT images with the automatic method showing robustness in difficult cases, including: (**A**) Thick calcium (GA) and near-wall blood residue (YA); (**B**) Fuzzy guidewire artefacts near the lumen boundary (GA) and side branch outside the main vessel wall (YA); (**C**) Changes in bright/dark tissue patterns at the outer boundary (GA) and side branch within the artery wall; (**D**) Lipidic (YA) and fibrous tissue (GA); (**E**) Side branch close to the outer wall (GA) and blood contrast near the lumen (YA); (**F**) Discontinuous outer wall (YA) segmentation still closely resembles expert annotation (GA); (**G**) Lipidic (YA) and fibrous thickening of the artery wall (GA); (**H**) Significant blood artefacts from improper flushing (both arrows); (**I**) Side branch connecting to the wall region (YA) and catheter touching the lumen wall (GA). Reprinted from [110], with permission, under the Creative Commons. YA = yellow arrow; GA = green arrow.

By extracting further information on vascular tissue components through polarization-sensitive OCT (PS-OCT) [107,108,109], Haft-Javaherian et al., were able to detect the lumen, intima and medial layers with impressive absolute distance errors of 2.36 ± 3.88 µm, 6.89 ± 9.99 µm and 7.53 ± 8.64 µm, respectively (Figure 6) [110]. Comparisons between the automated approach (blue) and expert annotation (red) showed strong ability to handle many difficult, yet common, features observed in OCT pullbacks. Carried out on a small dataset of 984 images (from 57 patients), a multi-term, multivariate loss function was created through combination of five common functions, namely: dice; weighted cross-entropy; topological; boundary precision loss; and an attending physician loss function to account for manual input. When applied through a U-Net based deep residual learning model using a leaky rectified linear unit (ReLU) function [111], overall classification accuracy for six components were: plaque shadow 0.82, guidewire shadow 0.97, lumen 0.99, intima 0.98, media 1.00 and outer wall 0.99. This approach could also be useful in segmenting the outer elastic membrane in hybrid IVUS-OCT systems [112], where the multivariate loss function could manage the added information provided by IVUS while maintaining the high-resolution OCT image characteristics during segmentation. Although showing impressive accuracy, the segmented outer boundaries in this approach did not always produce smooth contours, particularly in diseased regions where signal attenuation was high (see Figure 6A,D,F–I).

**Figure 7:**
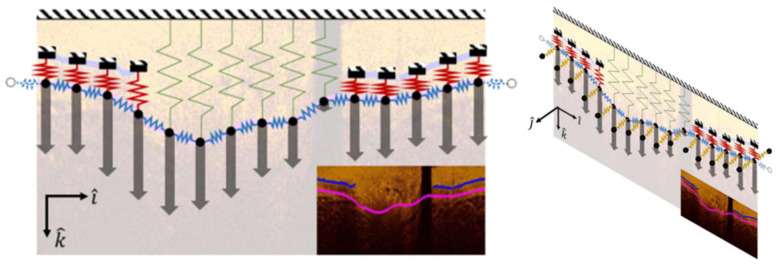
Outline of the surface fitting technique using four different spring stiffnesses (blue, green, yellow, and red) fitted either to visible sections of the outer elastic membrane or the detected lumen contour. Nodes (black circles) were connected to adjacent nodes within the image frame as well as both proximal and distal frames. Gray arrows represent the applied forces proportional to the sum of A-line pixel intensities. The surface fitting and force-balance optimization was carried out across the entire pullback (*j* direction) to generate a smooth and continuous outer wall over the entire artery section. © [2019] IEEE. Reprinted, with permission, from [113].

Discontinuous contours produce challenges when applying results to 3D modelling (in both computer-aided design (CAD) or finite element mesh (FEM) packages) and do not represent biological tissues well. Addressing this challenge, Olender et al., developed a 3D surface fitting technique using a mechanical, spring based approach [113]. This method was designed to ensure smoothness of the outer wall over the entire pullback through a force-balance/constrained nonlinear optimization method. By using edge detection methods to segment the outer elastic membrane in healthy wall regions and fitting of an anisotropic, linear elastic mesh to the associated A-line locations, forces proportional to the sum of A-line pixel intensities were then added (Figure 7) [114]. The resulting iterative force-balance optimization resulted in a mean difference in area (MADA) of 0.93 ± 0.84 mm^2^ compared to expert annotation in 724 images from seven patients. Further validation against manually annotated and co-registered IVUS pullbacks resulted in a MADA of 1.72 ± 1.43 mm^2^ (19.2 ± 15.0%). While surface smoothing and fitting times were 2.74 ± 0.28 ms and 40.20 ± 7.50 ms per frame, respectively, this approach would benefit from improvements to the lumen and edge detection speeds which required a much greater 4.20 ± 1.50 s and 5.35 ± 0.85 s per frame, respectively, to make it clinically applicable. This approach shows promise for smoothly segmenting the outer wall in OCT images while constraining atherosclerotic tissue classification approaches.

## 4. Plaque Characteristics and Subtypes

**Figure 8:**
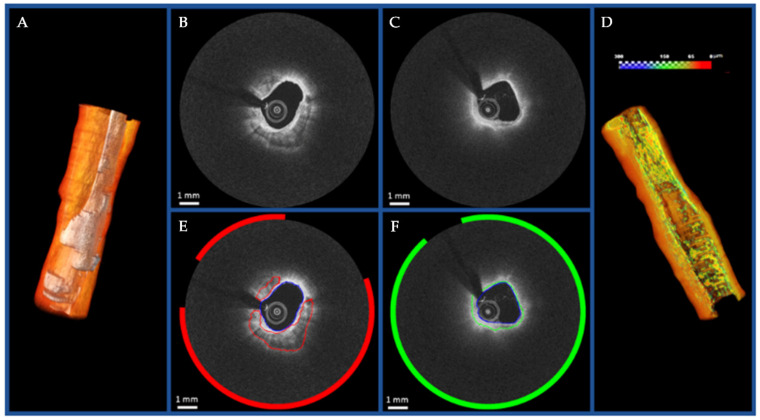
Visualization of the proof-of-concept automated segmentation and 3D rendering results for calcific (**A**) and lipidic (**D**) plaques. The original images and the corresponding automated segmentation for calcific lesion and fibrous cap over the lipid component are shown in (**B**,**E**) and (**C**,**F**), respectively. Reprinted from [115], with permission, under the Creative Commons.

Finding critical features to help accurately classify coronary plaques is an important research focus, as computation time is heavily dependent on the number of plaque features acquired. These morphological features, including optical characteristics, lumen morphology, A-line peaks and texture analyses were further investigated in [115]. Here a three-class random forest (3C-RF) classifier was compared to a similar three-class support vector machine (3C-SVM) as well as a dual binary (DB) classifier; the difference being the three-class classifiers simultaneously searched for fibro-calcific and fibro-lipidic A-lines, whereas the DB followed a sequential approach. Using both the minimal-redundancy-maximal relevance (mRMR) [116] and binary Wilcoxon [117] methods combined with conditional random field (CRF) [118] denoising, a total of ten feature selection and classification schemes were tested on a dataset of 6556 images (49 pullbacks) and histologically validated on 440 ex vivo images (10 pullbacks). It was found that lumen morphology and 3D edge/texture features from the Leung-Malik filter bank [119] provided the largest improvements in classification accuracy of up to 81.6% in the 3C-SVM with mRMR feature selection. This segmentation was then translated into a 3D rendering to demonstrate an automated, proof-of-concept segmentation tool, shown in Figure 8.

However, Zhang et al., demonstrated that a fully convolutional DenseNet based classification network with up sampling path for resolution restoration outperforms both SVM and U-Net based CNN architectures in fibrous cap thickness quantification. A critical measure of plaque stability, respective fibrous cap thickness errors of 13.06%, 22.20% and 17.46% were shown [120,121,122]. These errors are due to the high signal attenuation and diffuse contours representative of a fibrous cap overlying a lipid pool coupled with inter-observer variability and expert interpretation in the manually segmented ground truth. As accurate thickness measurement is a critical parameter for quantification of plaque vulnerability and biomechanical stress, further research to address these challenges and reduce errors is required [123]. Techniques such as dynamic programming have also demonstrated the capability to overcome these challenges and could be further explored [124,125]. This study was also limited to using only 1008 images (after data augmentation) from two patients, suggesting room for larger, more detailed studies in the future.

Further developments have also been made in automatically differentiating between a larger number of atherosclerotic tissue types [92,126,127,128,129,130,131,132,133,134,135,136]. Beginning with fibrous plaques, Wang et al., proposed a hybrid mix of a gaussian mixture model (GMM) and fourth-order nonlinear partial differential equation (PDE) which extended an adaptive diffusivity function to overcome the challenges that classical GMMs face in noisy images [128,137]. The method significantly outperformed five other algorithms under ongoing research: (1) FRSCGMM—fast and robust spatially constrained Gaussian mixture model [138]; (2) AFPDEFCM—fourth-order PDE-based fuzzy c-means [139]; (3) FCM—PDE-based fuzzy c-means [140]; (4) SMM—Student’s-t mixture model [141]; (5) standard GMM [142]; and _6) GMM-SMSI—GMM with spatial pixel relationship extracted using a saliency map [143]. Further improvements were presented in fibrotic plaque detection by Liu et al., who demonstrated that a CNN based on the VGG-16 network outperformed the single-shot detector (SSD) and you only look once (YOLO)-v3 based models, with accuracies of 94.12%, 93.75%, and 64.89%, respectively [144,145,146,147,148,149]. However, a more significant challenge is differentiating fibrous from other plaque classifications [45].

To assess the vulnerability of plaques, quantifying multiple plaque components and subtypes is essential. Liu et al., developed an ensemble method to combine the outputs of multiple networks to improve the accuracy of detecting vulnerable regions [150]. By combining the Adaboost, YOLO, SSD, and Faster region-based CNN outputs, a precision and recall of 88.84% and 95.02%, respectively, were reached, with a total detection quality of 88.46%. To further improve vulnerable plaque assessment, Gerbaud et al., introduced an adaptive attenuation compensation algorithm to assist in visualizing the outer elastic membrane in in regions of high attenuation [151]. This allowed plaque burden to be quantitively and automatically assessed, resulting in a mean difference of 0.27 ± 3.31 mm^2^ for the outer elastic membrane and −0.5 ± 7.0% for plaque burden when compared to matched IVUS frames. Such capability overcomes one of the most significant limitations associated with OCT use and could be further used to assist quantifying the lipid core burden index proposed in [152]. By further developing a normalized-intensity standard deviation (NSD) measure, Rico-Jimenez et al., were also able to successfully automate the detection of macrophage infiltration in regions of intimal thickening, fibrous plaque and fibroatheroma, resulting in an accuracy, sensitivity and specificity of 87.45%, 85.57% and 88.03%, respectively, in a k-fold validation against manual segmentation [153]. Through the introduction of a pyramid parsing network, with encoder consisting of a ResNet50 based CNN, Shibutani et al., were also able to detect regions of previous rupture/erosion that have since healed [154]. The ex vivo assessment and histological comparison of 1103 segments showed excellent area under the curve of 0.86, highlighting the potential for future automated classifiers to recognize emerging risk factors.

A key focus has been the classification of atherosclerotic tissue into fibro-calcific and fibro-lipid components through A-line characteristics [115,155,156,157]. Kolluru et al., showed that CNN classification more closely resembled expert annotations than an ANN, despite similar accuracy for both fibro-calcific and fibro-lipid components [155]. With this knowledge, Lee et al., compared the classification accuracy of the SegNet and Deeplab v3+ CNNs [157,158,159]. The 91 layered SegNet network, pre-trained in the ImageNet dataset [160], outperformed the Deeplab v3+ network for both fibro-lipidic (Dice: 0.83 ± 0.06 vs. 0.780 ± 0.077; Jaccard: 0.73 ± 0.073 vs. 0.65 ± 0.10) and fibro-calcific (Dice: 0.90 ± 0.04 vs. 0.82 ± 0.07; Jaccard: 0.83 ± 0.04 vs. 0.70 ± 0.10) A-line classifications, respectively. Investigations have also suggested that including attenuation coefficients in A-line classification of fibro-calcific and fibro-lipid components can further increase accuracy, including differentiation from other tissue types (mixed, macrophages, necrotic cores) [161,162,163]. The network architecture totaled five pooling/unpooling layers with 26 convolutional layers and added image padding to avoid misclassification due to edge effects. This architecture was then applied in a hybrid learning approach on 6556 images from 49 patients with a RF classifier [156] implemented due to the faster computation time, needing only 25% of the training time and 33% run time of a SVM to achieve comparable accuracy. When a CRF was applied for noise postprocessing, the hybrid model approach outperformed a purely CNN for fibro-calcific (sensitivity: 97.20% vs. 80.20%; specificity: 91.90% vs. 92.90%) and fibro-lipid (sensitivity: 77.30% vs. 46.80%; specificity: 91.90% vs. 92.90%) classification, needing approximately one second per image (the majority, 0.9 s, required for feature extraction). The key differentiator here was that the hybrid method made use of morphological features.

To investigate the classification of fibrous tissue alongside calcification, macrophages, neovascularization and healthy intima/media layers, Abdolmanafi et al., compared three CNN based feature generators (AlexNet [164], VGG-19 [145] and Inception-v3 [165]) to train a RF classifier [132]. Although features generated from pre-trained networks are useful to reduce training/computation time, results show that accuracy, sensitivity, and specificity suffer when supervised fine tuning is not applied. To overcome this, a weighted majority voting approach was applied to the RF results from each set of features, leading to significant improvements in performance over 33 patients (Accuracy: 0.99 ± 0.01%; Sensitivity: 98.00 ± 2.00%; Specificity: 100.00 ± 0.00%). This method outperformed an FCN trained on a larger 5040 image (45 pullback) dataset [133]. By making use of dilated convolutions for semantic segmentation and spatial pyramid pooling modules, Abdolmanafi et al., further developed an FCN capable of classifying and segmenting tissues into fibrous, fibro-calcific, fibroatheroma, thrombus, and micro-vessels with accuracy of over 93% in each case [134]. They demonstrated that the ADAM optimizer and weighted cross-entropy loss function outperformed stochastic gradient descent and the dice loss coefficient, respectively, in the 41-pullback dataset. While ADAM in particular may outperform stochastic gradient descent, its generalization performance may suffer, hampering translation to other datasets [166]. Interestingly, this approach also made use of the original image rather than A-lines from the polar transform, reducing the computational cost associated with this pre-processing step whilst maintaining accuracy.

**Figure 9:**
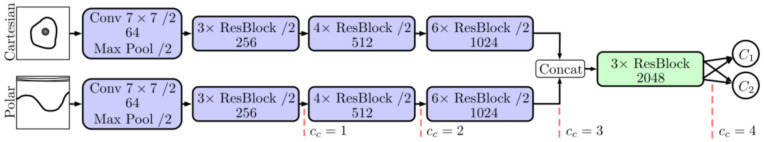
Layout of the dual-path ResNet model for automated extraction, making use of both the cartesian and polar image representations. Points *Cc* represent varying concatenation locations which were assessed for the two paths. © [2019] IEEE. Reprinted, with permission, from [130].

Polar and cartesian representations of OCT images can provide varying features for automated extraction. This was exploited by Gessert et al., with a multi-path architecture, as shown in Figure 9 [130]. Variations in concatenation points for feature fusion, transfer learning approaches and data augmentation resulted in an overall best performance of 91.70%, 90.90%, and 92.40% for accuracy, sensitivity, and specificity, respectively (F1 score of 0.913) [130]. The dual path variations of ResNet-v2 [97] and DenseNet with late feature concatenation increased accuracy by 1.4% and 1.8%, respectively, suggesting some added benefit from combining features from cartesian and polar image forms. Interestingly, cartesian based images saw a more significant gain in accuracy with both data augmentation (16%) and transfer learning approaches (15%), compared to polar images. Both methods were shown to outperform other models to classify vulnerable plaque when applied to a deep residual, U-Net based CNN [126,135]. The traditional encoder was replaced with the pre-trained ResNet101 for transfer learning improvements while rotational based data augmentation increased the number of images ten-fold (to 8000). A multi-term loss function was proposed to overcome imbalances in foreground/background pixels, which can lead to incomplete vulnerable region detection. By combining the weighted cross-entropy loss function, to enhance boundary pixels and improve boundary segmentation, and dice coefficient, to increase pixel classification accuracy, an overall pixel accuracy and precision of 93.31% and 94.33%, respectively, were reached [135], improvements of 49% and 14%, respectively, over the initial prototype U-Net. More impressively, the mean intersection over union and frequency weighted intersection over the union, improved measures of the overlap in two regions, improved 103% and, 71%, respectively.

Calcified plaques generally present more favorable optical properties for segmentation [45]. Using a deep CNN, trained on the ResNet-50 network over a dataset of 4860 images (18 pullbacks), He et al., managed a precision, recall and F1 score of 0.97 ± 0.01, 0.98 ± 0.03, and 0.96 ± 0.03, respectively [167]. This result was achieved by the zero-padding, 3D ResNet network trained in the ImageNet dataset making use of the ADAM optimizer, which outperformed the same network setup for the 2D ResNet. Here, data augmentation was also shown to be an important step, reducing model overfitting, and strengthening the generalizability. In comparison, using a U-Net based architecture with the same binary cross-entropy loss function, Avital et al., managed an impressive accuracy of 0.99 [168]. However, this classification and segmentation still requires translation to 3D geometries for the purpose of application in biomechanical simulation.

**Figure 10:**
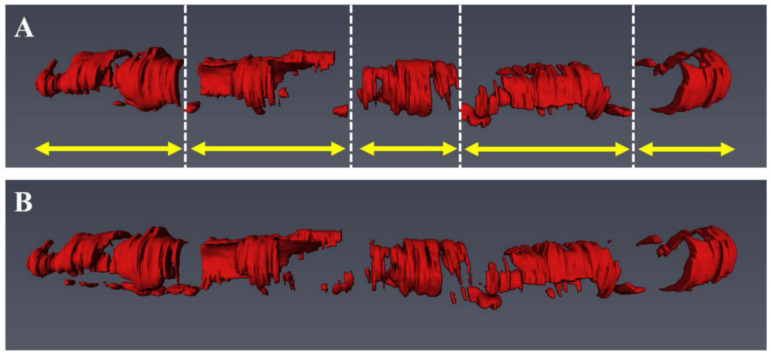
Visualization of the five major calcified lesions (yellow arrows) after 3D reconstruction and comparison between the manually annotated ground truth (**A**) and the automated method (**B**). Reprinted from [169], with permission, under the Creative Commons.

Building on their previous work, Lee et al., developed a two-step process to both segment and reconstruct 3D calcification models, as shown in Figure 10 [169]. Here a deep learning CNN model was used for classification followed by the pre-trained SegNet network developed in [170]. The initial classification made use of transfer learning from the VGG-16 and VGG-19 networks with five-fold cross validation and final use of the Tversky loss function, which provided superior performance compared to the weighted cross-entropy and dice loss coefficients. Importantly, a fully connected CRF was applied to denoise the output and create labels with more relevant spatial characteristics, an important step for 3D reconstruction. This resulted in calcification detection sensitivity, specificity and F1 score of 97.70%, 87.70%, and 0.92, respectively, from a dataset of 8231 images (68 patients). This improved upon earlier sensitivity and dice coefficients of 85.00 ± 4.00% and 0.76 ± 0.03 [170], respectively, from a one-step, weighted VGG-16 based CNN that was tested on 2640 images from 34 pullbacks and trained on the CamVid dataset [171]. Furthermore, the two-step approach reduced misclassification of tissues adjacent to calcifications, resulting in more accurate calcification angle, depth and thickness measurements and subsequently better segmentations. Of note, at least 3900 images were required for training of the two-step method to obtain stable and reproducible results, highlighting the need for larger, expert annotated datasets.

Dealing with limited datasets, with either scarce or weak annotations, is a significant challenge in the medical field and an ongoing research focus [55]. Rather than addressing the challenge of dataset size by building larger datasets, Kolluru et al., proposed to reduce the number of images needing expert annotation [172]. By focusing on calcified lesions, a deep feature-based clustering technique was developed to identify images needing expert annotation from identified volumes of interest (VOI). This removed the need to manually annotate a complete set of training labels, reducing a significant time cost. The clustering method was compared to annotation of equally spaced images on a dataset of 3741 images (60 VOIs from 41 pullbacks), outperforming the equally spaced annotation dataset using just 10% of the total selected images. Further development and use of approaches such data augmentation, transfer, and active learning, CRF post-processing and class activation mapping to reduce the number of annotated images needed for accurate training and classification would benefit the field.

## 5. Stents

OCT can be used both immediately after stent deployment to visualize stent sizing, apposition of struts against the intimal surface and to identify acute stent-related complications (e.g., stent-edge dissection). Furthermore, it also plays a role when assessing the underlying nature of later stent complications, such as in-stent restenosis caused by neointimal hyperplasia or neo-atherosclerosis and stent thrombosis. The automatic detection, segmentation and quantification of stent strut mal-apposition post stent deployment could assist in analyzing areas at increased risk of subsequent neointimal proliferation, stent thrombosis and MACE [173]. Early classification of this apposition and neointimal coverage was carried out using a supervised ANN on a relatively small dataset of 20 pullbacks [174]. Twenty-two A-line features in polar coordinates were extracted based on image intensity gradients in similar fashion to early lumen-based segmentation, but with the addition of strut shadow gradients to classify candidate regions of interest (ROI). A-line representation (previously visualized in Figure 1) of stent struts and their shadows were suggested to be less affected by artefacts and rotational distortion in polar coordinates, a preferable characteristic for automated classification [175]. Based on a split of 70%, 15% and 15% split for training, validation, and testing, respectively, results showed a strong positive predictive value of 95.60% (97.40% vs. 95.10% for uncovered and covered struts, respectively). However, these results were influenced by image quality, with covered struts in particular suffering from a lower positive predictive value of 86.10% in suboptimal image sets.

To improve stent strut segmentation in suboptimal images, such as those with residual blood artefacts, Cao et al., investigated an AdaBoost trained, cascade classifier [176]. With a combination of three filters of varied angles developed through a dynamic programming approach, true positive scores of 0.87–0.93 in image sets with significant blood artefacts (F score 0.88–0.89) were achieved, comparable to images without artefacts (TPR 0.91–0.96; F score 0.90–0.93). While still using a relatively small dataset of 15 pullbacks (4065 images and 12,550 struts), the overall recall rate for covered struts was 0.98. The resulting malapposition calculation matched well with manual segmentation, although with a slight increase due to the false positive rate of 26.70% driven by images with significant blood artefacts.

Another challenge presented in stented arteries is variation in the optical characteristics between bare metal stents (BMS) and bioresorbable vascular scaffolds (BVS). While metallic stents present with well-defined edges and an invisible strut backside/pronounced shadow, BVS edges are well defined around a dark core [177]. Focusing on metallic stents, Jiang et al., compared the performance of the YOLOv3 framework and a region-based fully-convolutional neural network (R-FCN) [178]. The YOLOv3 framework made use of a binary-cross entropy loss function and K-means adjusted anchor box detector using the SSD method, while the R-FCN combined log-classification and smooth regression loss functions and a novel position-sensitive feature score map. Although obtaining similar results, the R-FCN eventually reached the highest precision of 99.8%, although the test set consisted of only 425 images. In contrast, Amrute et al., built on previous work to automatically segment BVS using an unsupervised K-means clustering approach [179]. A positive predictive value of 93.00% was reached through testing on 1140 images. Building on this work, Lau et al., focused on segmenting both BMS and BVS with one architecture [180]. The MobileNetV2 [181] was first combined with the U-Net architecture to reduce computational cost and compared to the DenseNet121 encoder, with the overall best dice coefficient of 0.86 for the segmentation of the BVS. However, misclassification of images with bright fringes (common in BMS), dark shadowing, fractured struts, and areas of large neointimal coverage is common in many approaches. These are still future challenges to be overcome for automatic strut detection methods.

**Figure 11:**
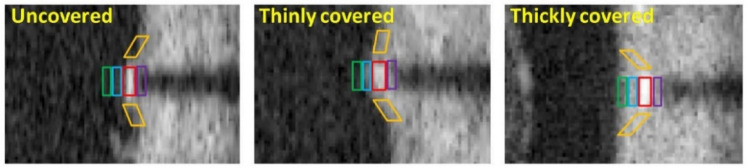
Patches used to extract features for uncovered, thinly covered, and thickly covered struts. Side patches (orange) capture continuity of the tissue, while the green, blue, red, and purple patches highlight the front, middle, stent strut and backside pixel regions, respectively. Reprinted from [182], with permission, under the Creative Commons.

By building larger datasets for training and validation, Lu et al., further addressed the challenges of stent apposition, quantitative coverage measurement and detection in regions of strut clustering [182]. In 80 pullbacks (7125 images) with 39,000 covered and 16,500 uncovered struts, 21 features (including patch features shown in Figure 11) were chosen through a forward feature selection technique with a bagged decision trees classifier. By using a SVM for classification (LIBSVM library [183]) and a graph-based mesh growing technique to overcome challenges associated with stent struts that were clustered close together, a sensitivity and specificity of 94.00 ± 3.00% and 90.00 ± 4.00%, respectively, were obtained. This approach was further developed into a toolkit (OCTivat-Stent), published in 2020, capable of reducing total segmentation time to just 30 min per pullback, from 6–12 h through manual annotation [184]. Additionally, specificity was greatly improved as strut coverage increased beyond 40 µm, with further research needed to accurately and consistently quantify thinner neointimal coverage.

**Figure 12:**
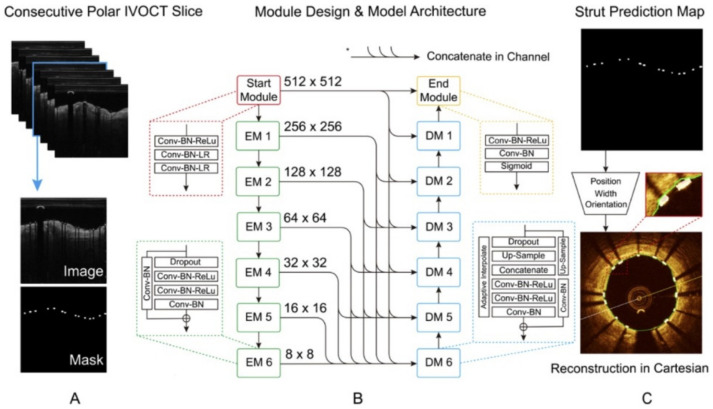
Layout of the presented model for stent strut segmentation. (**A**) The pseudo-3D polar image stack and manually annotated strut mask were taken as inputs. (**B**) Strut segmentation model composed of a start module, six encode and decode modules and an end module. (**C**) The predicted strut map including orientation, width, and position of struts. Reprinted from [175], with permission, under the Creative Commons.

Feature-based segmentation still encounters challenges with varying acquisition settings and patients, as well as difficulty translating between stent designs without manual intervention. With this in mind, Wu et al., developed a CNN architecture based on the U-Net and RefineNet architectures [185] (Figure 12), to segment stent struts from pseudo-3D image stacks in polar form [175]. The pseudo-3D form uses prior knowledge of the implanted stent design and consecutive image slices to constrain the segmentation results, similar to a previous approach for constraining the 3D segmented point clouds to known strut skeletons [186]. The four-stage deep CNN architecture, consisting of start and end modules sandwiching the encoder and decoder, made use of batch normalization and convolution operations to mitigate gradient degradation and shortcut connections to minimize loss of spatial resolution, common factors impacting strut detection. With 80% of images used for training with the ADAM optimizer and combined binary cross-entropy and Tversky loss functions over 300 epochs, the deep CNN outperformed all feature-based techniques as well as the same deep CNN without the pseudo-3D image input. This highlights the importance of using consecutive image slices and prior knowledge of the stent structure to classify and detect struts. Importantly, in a dataset of 170 pullbacks (205,513 stent struts) containing 13 stent designs, overall results for dice coefficient, Jaccard index and precision were 0.91 ± 0.04, 0.84 ± 0.06 and 0.94 ± 0.04, respectively, highlighting the ability of this approach to handle difficult cases of malapposition and intimal coverage.

**Figure 13:**
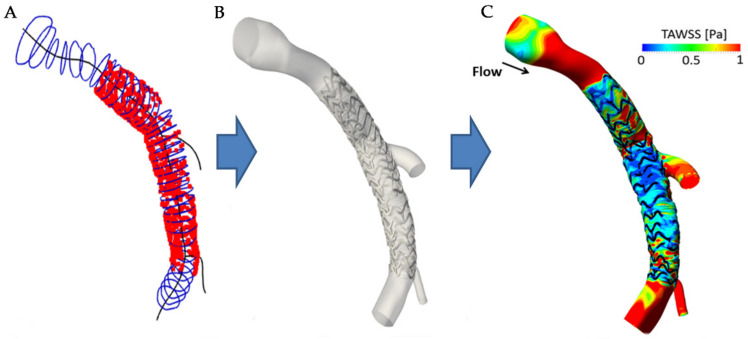
Automatically generated 3D stented artery model. (**A**) OCT contours (blue) and stent struts (red) placed along the 3D centerline (black). (**B**) Generated 3D surface model. (**C**) Wall shear stress resulting from CFD simulation. Reprinted from [64], with permission, under the Creative Commons.

Application of these segmentation methods to computational simulation requires the additional step of 3D reconstruction of both the stent structure and lumen surface. Building from in vitro models with application of the Sobel edge detection and interpolation between detected struts [187,188], Migliori et al., used a fuzzy logic approach for classification of a Multi-link 8 stent (Abbott Laboratories, Abbott Park, IL, USA) and subsequent 3D reconstruction with reasonable agreement to manual approaches [189]. To improve the stent reconstruction, Elliot et al., made use of diffeomorphic metric mapping to develop a constrained iterative deformation process that configures an initial undeformed stent geometry to the 3D imaged point cloud [190]. Tested on two stents (Integrity bare metal stent and Xience Alpine drug eluting stent) in four in vitro models and compared to manual segmentation and reconstruction, results showed good agreement, with an average distance between the strut centroids of 97.5 ± 54.4 µm. In in vivo cases, by improving lumen segmentation around struts with a novel correction step to account for blood artefacts, Bologna et al., automatically generated a stented artery model for simulation of WSS from the OCT based 3D point cloud and biplane angiography centerline (Figure 13) [64]. However, these approaches suffered in the case of struts that did not have visible, continuous, or square outlines. Building on this with an enhanced reconstruction method using prior knowledge of the undeformed stent geometry, O’Brien et al., automatically analyzed four swine models using attenuation coefficients and a decision tree classifier, expanding previous studies to obtain good agreement with manual segmentation [186,191,192]. WSS results from the enhanced simulation showed improved resolution in the hemodynamic microenvironment compared to the unenhanced method. Furthermore, a strong association between WSS and strut-lumen distance was seen, highlighting the importance of accurate classification, segmentation, and reconstruction for 3D simulation results.

## 6. Discussion

Methods to automate the classification and segmentation of pathological and non-pathological formations in intravascular OCT images are emerging as clinically feasible. To automatically segment the lumen, the deep capsules approach presented by Balaji et al., showed impressive accuracy, speed and efficient computational use which make it an ideal candidate to make it to clinical use [93]. This approach built upon the useful characteristics of the U-Net to maintain high-level feature accuracy and shows strong promise to be expanded to plaque component analysis. However, this approach should also be expanded to be able to segment bifurcation regions and requires further work to better handle fringe cases (i.e., increasing the number of cases with artefacts and difficult geometries). Addressing the artery layers and outer wall, the mechanical approach presented by Olender et al., demonstrated impressive speed when fitting and smoothing a 3D surface from all images in a pullback [113]. This overcomes OCT’s most significant limitation, penetration depth in deep atherosclerotic components. However, its lumen and outer elastic membrane identification speed still lacks and could benefit from the U-Net based network proposed by Haft-Javaherian et al. [110]. This approach could also show promise for automating the segmentation of tissue in future hybrid imaging modalities, such as a combined IVUS-OCT probe [193], as its multivariate loss function could manage the added information that IVUS presents. Various techniques provided strong segmentation capability for plaque compositions and coronary stents, with CRF de-noising and strut detection constraints with prior knowledge of stent design more critical factors than the underlying network to providing strong results. However, further research is required to target quantifying fibrous cap thickness accurately in image datasets that well represent real-world scenarios, with current studies significantly limited to small datasets (179–348 images in each study to date [123,124,125]). Until studies have access to datasets that are representative of real-world scenarios, clinical application will remain limited.

Furthermore, while these methods show strong promise, assessing their effectiveness is not a straight-forward task, as heterogeneity in evaluation metrics can lead to an incomplete assessment of a methodology. A wide range of evaluation metrics have been used to assess the performance of automated techniques, with significant research applied to developing distance, similarity and boundary overlap metrics [194,195]. Choosing the most effective measure for the task at hand is difficult and can lead to bias in results, particularly when dealing with class imbalance [196]. Making use of frequency weighted evaluation metrics, such as the frequency weighted intersection over union rather than the commonly used Jaccard similarity index could assist in dealing with this challenge. Development of consensus documents for OCT based deep learning may also assist researchers reduce other biases in their work, including data distribution, dataset leakage and methodological bias, factors already shown to significantly skew results in cancer diagnoses [197,198,199,200]. Improving access to large scale, longitudinal and multicenter datasets that are representative of real-world scenarios coupled with consistent use of techniques including cross-validation, model regularization (to prevent overfitting or underfitting) and de-biasing through oversampling and adversarial de-biasing will help in addressing these challenges. Competitions, such as [201], could further assist by standardizing the development and evaluation of methods on pre-defined datasets, improving transparency, while open-source projects, such as the medical open network for artificial intelligence (MONAI), first publicly released in 2020, provide best practice deep learning frameworks [202].

Reviewed studies primarily used supervised learning techniques, such as neural networks, RF and SVM, where the model has access to both the original image, as well as manually annotated versions during training to effectively learn the correct parameters [85,101,156]. This requires large, high-quality, manually annotated datasets for training and validation to produce accurate and robust results, a significant cost. A focus on addressing this challenge by handling imperfect datasets with sparse or no manual annotations is emerging [55]. State-of-the-art unsupervised learning techniques, such as generative adversarial networks (GAN) and autoencoders, are also gaining in popularity and could reduce this burden by learning patterns from unlabeled data or generating further image labels to optimize segmentation [203,204]. While Abdolmanafi et al., applied a sparse autoencoder in their work segmenting atherosclerotic tissue types [134], recent advancements in autoencoders applied to CT imaging are also leading to stronger feature learning and dimensionality reductions that could translate for use in intravascular OCT [205].

**Figure 14:**
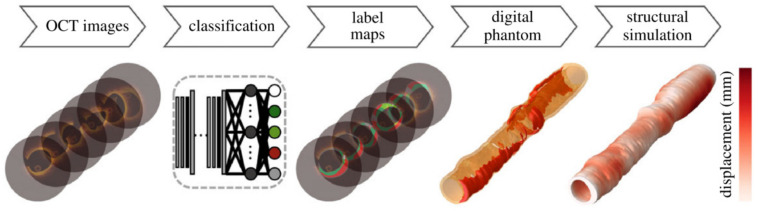
Framework layout for the automated reconstruction and 3D structural simulation of an artery. Initial OCT images were stacked to form a pseudo-3D image sequence before classification with a CNN and generation of label maps which were subsequently smoothed into contours to generate the digital phantom which was converted to a finite element mesh for structural simulation. Republished with permission of The Royal Society Publishing, from [206]; permission conveyed through Copyright Clearance Centre, Inc.

With improvements in classification and segmentation capability, there is a growing need to integrate these advances into automated 3D reconstructions in a sufficient framework for biomechanical simulation. Lumen and stent-based investigations have already begun developing this ability for clinical application [91,93]. However, structural based analysis still lags due to the added complications of generating smooth and sufficiently connected regions for finite element mesh generation. To the best of our knowledge, the only framework to integrate image classification, segmentation, 3D reconstruction and structural simulation is that recently presented by Kadry et al. [206]. This framework, shown in Figure 14, built on their previous works to classify pixels into six tissue components within a constrained wall area region, making use of 3D mode filtering to improve spatial consistency and continuity of contours [113,114,131]. This approach shows significant potential to translate to clinical use, as it brings together the relevant processing steps into a single framework. Future work could also be made to account for motion artefacts within intravascular imaging, which were suggested to result in relative stenosis length errors of up to 160% (compared to 0.6% after motion catheter trajectory and time synchronization) [207]. While an impressive step forward, future work is still required to integrate an imaging modality capable of generating an accurate 3D centerline to stack the 2D contours [208,209,210,211]. Of the available modalities that could be used, invasive coronary angiography is the primary candidate due to its widespread clinical use and requirement during intracoronary OCT procedures. However, computed tomography coronary angiography is a rising noninvasive contender and coronary magnetic resonance imaging could also be a useful addition to reduce patient radiation and contrast exposure, although lower image resolution and susceptibility to motion related image degradation could impact reconstruction accuracy in these cases [212,213].

Multi-modal intravascular imaging modalities also have the capability to further overcome challenges with automatic OCT segmentation. The integration of OCT and IVUS, for example, could overcome the limited 0.1 to 2 mm penetration depth associated with OCT in plaques, removing the need for complex estimation techniques to segment the outer wall or plaque backsides and quantify plaque burden in regions of high attenuation [193,214]. The complementary capabilities of these two imaging modalities have already demonstrated their potential to increase positive predictive capability when detecting TCFA [215]. Developments in OCT also show promise for providing useful histopathological information, with PS-OCT [108] demonstrating incremental value in the segmentation of artery layers and the outer wall [110]. Furthermore, molecular information obtained from multi-modal imaging could assist in automatically segmenting emerging vulnerable features, such as layered plaques, indicative of previously destabilized plaque that has since healed, or collagen arrangement within the fibrous cap, which could suggest lesion instability [216,217]. Further development of near-infrared spectroscopy/Raman, fluorescence lifetime (FLIM) and near-infrared autofluorescence (NIRAF) modalities in combination with OCT also shows promise to extract biochemical and molecular tissue information on elastin and macrophages whilst nuclear imaging techniques such as positron emission tomography (PET) could supplement this with information on local inflammatory responses [112,218,219,220].

This molecular imaging capability could lead to more accurate classification and segmentation of vulnerable plaque regions. For example, the first in-human study on NIRAF combined with OCT showed NIRAF associated with high-risk plaque phenotypes, complementing the structural information available through OCT [221]. Further advancements could also assist in differentiating between healthy re-endothelization or fibrin drug eluting stent coverage, improving the ability to stratify risk of late stent thrombosis [222]. Combining this ability to accurately segment pathological borders and extract molecular information, reminiscent of an advanced virtual histology IVUS/OCT [223,224], presents opportunities to reverse engineer tissue constitutive models and adapt structural simulations to patient-specific conditions, currently a major limitation in the field of biomechanics [225,226,227,228,229,230,231,232,233,234]. However, there is still a need for further evidence to determine which multi-modal imaging technique can provide the strongest incremental benefits and risk stratification to improve both clinical outcomes and simulation capability.

Clinician acceptance of machine learning algorithms, especially in the case of intravascular OCT, is still tied to the imaging modality’s clinical utility. While OCT and IVUS are still not a part of routine coronary angiography procedures, automated segmentation approaches that can run in near real time in the catheterization laboratory could provide a significant advance in making quantitative data (e.g., fibrous cap thickness measurement) readily available to the interventional Cardiologist and assist with the interpretation of OCT images. In turn, this could inform clinical decision making and lead to better patient outcomes. The future potential for automated approaches to make it into clinical use also require addressing a number of systemic challenges, including: (1) Improving access to large scale, expertly annotated datasets to train and test techniques on data that is representative of real world scenarios; (2) Evidence that techniques are robust and reliable enough to enable clinical use and provide sufficient incremental value to justify the associated costs (i.e., health economic analysis); (3) Regulations surrounding the updates of medical technology could inhibit the rapid adoption required for AI in clinical scenarios; (4) Data ownership could impact how techniques develop, particularly if research techniques develop with large scale datasets to the point of commercial potential. [235]. These are both multi-disciplinary challenges and opportunities for the engineering, computer science and medical research fields.

## 7. Conclusions

Intravascular OCT is a high resolution, near-infrared light-based imaging modality capable of visualizing vulnerable plaque features, such as TCFA. Manual annotation of these images is a time consuming and tedious task, limiting its clinical application and use in 3D reconstructions for biomechanical simulation. With increases in computation power and numerical capability, automated techniques are emerging to classify and segment pathological and non-pathological formations, including vulnerable features. This review summarized recent advances (2016–2021) in automated techniques, applied to coronary OCT imaging and their subsequent application to 3D reconstruction and biomechanical simulation. Deep learning models have demonstrated the capability to classify and segment structural features in OCT imaging, including lipidic, calcific, and fibrous plaques, as well as stent and lumen borders in regions with considerable imaging artefacts. This capability is beginning to show potential for clinical use, with significant reductions in computation time allowing near real-time classification and segmentation. However, challenges surrounding access to large scale, expertly annotated image datasets that represent real-world scenarios and robustness of automated techniques to clinical use still need to be addressed before clinical acceptance. Further advances in multi-modal imaging catheters could increase the information available to automated techniques. When coupled with patient details and developments to streamline the process of 3D reconstruction and simulation, this capability could one day assist in guiding patient-specific care or intervention.

## Data Availability

Not applicable.

## Glossary of Performance Metrics

**Accuracy (ACC):** Accuracy is the proportion of pixels classified correctly out of the total number of pixels classified, defined as

(1) $$\mathrm{ACC}=\frac{\sum_{i=1}^{k}n_{ii}}{\sum_{i=1}^{k}t_{i}}\times 100,$$

where *k* is the total number of classification categories within the dataset, *t_i_* is the number of pixels belonging to the *i*th category and *n_ii_* is the number of correct pixel predictions of the *i*th category.

**Area under the curve (AUC):** Area under the curve determines an algorithm’s ability to distinguish between two classifications, with a value closer to one indicating better performance.

**Average symmetric surface distance (ASSD):** The average symmetric surface distance calculates the average distances, *D*, between point, *x*, on the boundary of the predicted region, ∂*P*, and its nearest point, *y*, on the boundary of the ground truth, ∂*GT*, and in reverse from the ground truth to the predicted surface.

(2) $$\mathrm{ASSD}=\frac{\sum_{x\in \partial GT}D(x,\partial P)+\sum_{y\in \partial P}D(y,\partial GT)}{\left|\partial GT\right|+\left|\partial P\right|},$$

**Bhattacharya distance (BHAT):** The Bhattacharya distance determines the similarity between two discrete probability density functions of image intensity values as [236]

(3) $$\mathrm{BHAT}=-\ln\left(\sum_{i=1}^{h}\left(\sqrt{P(i)Q(i)}\right)\right),$$

where *h* is the number of image intensity levels, in the case of image analysis, considered for the probability distributions *P* and *Q*.

**Coefficient of determination (R^2^):** The coefficient of determination defines how changes in a dependent or predicted variable are explained by changes in a second variable, described by

(4) $$R^{2}=\frac{\sum_{n}{\left(y_{n}-f_{n}\right)}^{2}}{\sum_{n}{\left(y_{n}-\bar{y}\right)}^{2}},$$

where there are *n* values of dataset *y*, and predicted values, *f*.

**Cohen’s kappa coefficient (CK):** Cohen’s kappa coefficient evaluates the reliability of agreement between two results, in this case the ground truth manual annotation and algorithm result, by taking into account chance and is evaluated as [237]

(5) $$\mathrm{CK}=\frac{p_{0}-p_{e}}{1-p_{e}},$$

where *p*_0_ is the observed agreement and *p_e_* is the probability of agreement by chance.

**Concordance-correlation-coefficient (CCC):** The Concordance-correlation-coefficient determines the agreement between variable *y* and a reference ground truth *x*, defined as [238]

(6) $$\mathrm{CCC}=1-\frac{{\left(\mu_{y}-\mu_{x}\right)}^{2}+\sigma_{y}^{2}+\sigma_{x}^{2}-2\rho \sigma_{y}\sigma_{x}}{{\left(\mu_{y}-\mu_{x}\right)}^{2}+\sigma_{y}^{2}+\sigma_{x}^{2}},$$

where the *µ_x_* and *µ_y_* are the variable means, *σ_x_* and *σ_y_* are standard deviations, *σ_x_^2^* and *σ_y_^2^* are the variable variances and *ρ* is Pearson’s correlation coefficient.

**Dice coefficient (DICE):** The Dice coefficient determines the overlap between two regions *A* and *B* as [239]

(7) $$\mathrm{DICE}=\frac{2\left|A\cap B\right|}{\left|A\right|+\left|B\right|},$$

where *A* is the region determined by the algorithm and *B* is the manually labelled ground truth or point of comparison, with higher values suggesting better performance.

**F1 score:** The F1 score compares the performance of two classifiers using their respective precision (PRE) and recall (REC) results, defined as

(8) $$F1=\frac{2\left(\mathrm{PRE}*\mathrm{REC}\right)}{\mathrm{PRE}+\mathrm{REC}}.$$

**False positive rate (FPR):** False positive rate is the ratio of false positives (FP) to false positives and true negatives (TN) combined, described as

(9) $$\mathrm{FPR}=\frac{\mathrm{FP}}{\left(\mathrm{FP}+\mathrm{TN}\right)}\times 100.$$

**Frequency weighted intersection over union (FIoU):** The frequency weighted intersection over the union determines the mean overlap between the algorithm calculated area and ground truth weighted by the frequency of occurrence of each category. Defined as

(10) $$\mathrm{FIoU}={\left(\sum_{i=1}^{k}t_{i}\right)}^{-1}\frac{1}{k}\sum_{i=1}^{k}\frac{n_{ii}}{t_{i}-n_{ii}+\sum_{j=1}^{k}n_{ji}},$$

where *k* is the total number of classification categories within the dataset, *t_i_* is the number of pixels belonging to the *i*th category, *n_ji_* is the incorrect prediction of the *j*th category when pixels belong to the *i*th category and *n_ii_* is the number of correct pixel predictions of the *i*th category.

**Hausdorff distance (HD):** The Hausdorff distance determines the largest of all the distances, *D*, between a point, *x*, on the boundary of the predicted region, ∂*P*, and its nearest point, *y*, on the boundary of the ground truth, ∂*GT*, defined as

(11) $$\mathrm{HD}=\max\left\{\underset{x\in \partial GT}{\max}D\left(x,\partial P\right),\underset{y\in \partial P}{\max}D\left(y,\partial GT\right)\right\}.$$

**Jaccard similarity (JS):** The Jaccard similarity index defines the size of the overlapping region divided by the size of the union of the two regions *A* and *B* as [240]

(12) $$\mathrm{JS}=\frac{\left|A\cap B\right|}{\left|A\cup B\right|}=\frac{\left|A\cap B\right|}{\left|A\right|+\left|B\right|-\left|A\cap B\right|},$$

where *A* is the region determined by the algorithm and *B* is the manually labelled ground truth or point of comparison, with higher values suggesting better performance.

**Kullback–Leibler divergence (KL):** The Kullback–Leibler divergence is a statistical distance measure evaluating the difference between two probability distributions, *P* and *Q*, over a domain of image intensity values, *h*, defined as [241]

(13) $$\mathrm{KL}=\sum_{i=1}^{h}P(i)\ln(\frac{P(i)}{Q(i)}).$$

**Mean average difference in area (MADA):** The mean average difference in area is calculated between the ground truth (*GT*) and predicted (*P*) result for *N* samples as

(14) $$\mathrm{MADA}=\frac{\sum \left(\left|GT-P\right|\right)}{N}.$$

**Mean intersection over union (MIoU):** The mean intersection over the union calculated the mean overlap between the algorithm calculated area and ground truth

(15) $$\mathrm{MIoU}=\frac{1}{k}\sum_{i=0}^{k}\frac{n_{ii}}{t_{i}-n_{ii}+\sum_{j=1}^{k}n_{ji}},$$

where *k* is the total number of classification categories within the dataset, *t_i_* is the number of pixels belonging to the *i*th category, *n_ji_* is the incorrect prediction of the *j*th category when pixels belong to the *i*th category and *n_ii_* is the number of correct pixel predictions of the *i*th category. This is also the mean Jaccard similarity index.

**Mean pixel accuracy (MPA):** The mean pixel accuracy determines the proportion of correctly classified pixels against the total number of pixels in each category, averaged across all categories,

(16) $$\mathrm{MPA}=\frac{1}{k}\sum_{i=0}^{k}\frac{n_{ii}}{t_{i}},$$

where *k* is the total number of classification categories within the dataset, *t_i_* is the number of pixels belonging to the *i*th category and *n_ii_* is the number of correct pixel predictions of the *i*th category.

**Misclassification ratio (MCR):** The percentage of misclassified pixels defined as

(17) $$\mathrm{MCR}=100-\mathrm{ACC}=⟮1-\frac{\sum_{i=1}^{k}n_{ii}}{\sum_{i=1}^{k}t_{i}}⟯\times 100,$$

where ACC is the accuracy defined earlier, *k* is the total number of classification categories within the dataset, *t_i_* is the number of pixels belonging to the *i*th category and *n_ii_* is the number of correct pixel predictions of the *i*th category.

**Negative predictive value (NPV):** The proportion of true negatives within the total negative algorithm predictions, defined as the ratio of true negatives (TN) to the sum of true negatives and false negatives (FN).

(18) $$\mathrm{NPV}=\frac{\mathrm{TN}}{\left(\mathrm{TN}+\mathrm{FN}\right)}\times 100$$

.

**Pearson’s correlation coefficient (R):** The population-based Pearson’s correlation coefficient for a pair of variables *X* and *Y* described by

(19) $$R=\frac{\mathrm{cov}(X,Y)}{\sigma_{X}\sigma_{Y}},$$

**Precision (PRE)/Positive predictive value (PPV):** The proportion of actual positives within the total positive algorithm predictions, defined as the ratio of true positives (TP) to the sum of true positives and false positives (FP).

(20) $$\mathrm{PRE}=\frac{\mathrm{TP}}{\left(\mathrm{TP}+\mathrm{FP}\right)}\times 100.$$

**Probabilistic rand index (PRI):** The probabilistic rand index measures the number of correct classifications made by an algorithm, using the true positive (TP), true negative (TN), false positive (FP) and false negative (FN) values, defined as

(21) $$\mathrm{PRI}=\frac{\mathrm{TP}+\mathrm{TN}}{\mathrm{TP}+\mathrm{FP}+\mathrm{FN}+\mathrm{TN}},$$

where *cov* is the covariance and *σ_x_* and *σ_y_* are standard deviations.

**Root mean square symmetric surface distance (RMSD):** The root mean square symmetric surface distance is defined as:

(22) $$\mathrm{RMSD}=\sqrt{\frac{1}{\left|\partial GT\right|+\left|\partial P\right|}}\sqrt{\sum_{x\in \partial GT}D^{2}(x,\partial P)+\sum_{y\in \partial P}D^{2}(y,\partial GT)},$$

and calculates the root mean value of all distances, *D*, between point, *x*, on the boundary of the predicted region, ∂*P*, and its nearest point, *y*, on the boundary of the ground truth, ∂*GT*, and in reverse from the ground truth to the predicted surface.

**Sensitivity (SEN)/Recall (REC)/True positive rate (TPR):** The proportion of true positive (TP) algorithm predictions out of the total actual positives predicted shown as the sum of true positive and false negatives (FN). In binary classifications, Sensitivity, Recall and True Positive Rate are equivalent.

(23) $$\mathrm{SEN}/\mathrm{REC}/\mathrm{TPR}=\frac{\mathrm{TP}}{\left(\mathrm{TP}+\mathrm{FN}\right)}\times 100.$$

**Specificity (SPE)/True negative rate (TNR):** The proportion of true negative (TN) algorithm predictions out of the total actual true negative predictions shown as the sum of true negative and false positives (FP).

(24) $$\mathrm{SPE}/\mathrm{TNR}=\frac{\mathrm{TN}}{\left(\mathrm{TN}+\mathrm{FP}\right)}\times 100.$$

## Appendix A

**Table A1:** Classified articles investigating automated coronary lumen segmentation. 3D—Three-dimensional. ACC—Accuracy. ADAM—Gradient based adaptive optimization. ASSD—Average symmetric surface distance. AUC—Area under the curve. BHAT—Bhattacharya distance. BR—Bifurcation region. CK—Cohen’s kappa coefficient. CNN—Convolutional neural network. DA—Data augmentation. DICE—Dice loss coefficient. FFR—Fractional flow reserve. HD—Hausdorff distance. IVUS—Intravascular ultrasound. JS—Jaccard similarity index. KL—Kullback–Leibler divergence. MADA—Mean average difference in area. MV—Main vessel NB—Naïve Bayes. NBR—Non-bifurcation region. NPV—Negative predictive value. OCT—Optical coherence tomography. PPV—Positive predictive value. R—Pearson’s correlation R^2^—Coefficient of determination. RF—Random Forest. RMSD—Root mean square symmetric surface distance. SEN—Sensitivity. SPE—Specificity. SVM—Support vector machine. TNR—True negative ratio. TPR—True positive ratio. WSS—Wall shear stress. * Expert annotation implies an experienced researcher carried out the annotation. Articles varied their use of manual segmentation and expert annotation and we match the description given in each article.

First Author [Ref]	Aim	Dataset	Morphological/Filtering Operations	Feature Detection/Classification	Outcome	Comparison *
Akbar et al. [65]	Automated lumen extraction and 3D FFR modelling	5931 images (40 patients)	Polar transform, Bilateral smoothing filter, dilation, erosion	L- & C-mode interpolation and Sobel edge detection	R: 0.99 FFR R: 0.98	Manual segmentation and individual L- and C-mode interpolation
Athanasiou et al. [91]	Lumen detection through optimized segmentation and 3D WSS modelling	11 patients, 613 annotated images	Polar transform, Bilateral smoothing filter	B-spline curve fit, K-means	3D HD: 0.05 mm (±0.19) R: 0.98 R^2^: 0.96 WSS R^2^: 0.95	Expert annotation and WSS results between expert annotated reconstruction
Balaji et al. [93]	Efficient and low memory automated lumen segmentation for clinical application	12,011 images (22 patients)	Gaussian derivative	PyTorch based deep capsules with ADAM optimizer	DICE: 0.97 ± 0.06 HD: 3.30 ±1.51 µm SEN: 93.00 ± 8.00% SPE: 99.00 ± 1.00%	Expert annotation, UNet-ResNet18, FCNResNet50 and DeepLabV3-ResNet50
Cao et al. [74]	Automated lumen segmentation in challenging geometries	880 images (five patients)	Polar transform, Narrow image smoothing filter (Gaussian)	Distance regularized level set	DICE: 0.98 ± 0.01	Manual segmentation
Cao et al. [76]	Automatic side branch ostium and lumen detection	4618 images (22 pullbacks)		Dynamic programming distance transform, differential filter	MV DICE: 0.96 BR DICE: 0.78 TPR: 0.83 TNR: 0.99 PPV: 87.00% NPV: 98.00%	Manual segmentation
Cheimariotis et al. [63]	Automated lumen segmentation in all image types (bifurcation, blood artefacts)	1812 images (20 patients, 308 stented, 1504 native)	Polar transform, Median filtering, Gaussian filtering, opening, Otsu binarization, low-pass filtering	Gradient window enhancement	Stented: DICE: 0.94 R^2^: 0.97 Non-stented: DICE: 0.93 R: 0.99 R^2^: 0.92	Expert annotation (area, perimeter, radius, diameter, centroid)
Essa at al. [70]	Automatic lumen detection in OCT (and tissue characterization in IVUS)	2303 images (13 pullbacks: Column-wise labelling 457, training 457, testing 1389)	Polar transform, A-line based dynamic tissue classification	Kalman filter based spatio-temporal segmentation method, RF	ACC: 96.27% HD: 11.01 ± 11.93 µm JS: 0.95 ± 0.03 SEN: 95.55 ± 3.19% SPE: 99.84 ± 0.29%	Expert annotation
Joseph et al. [68]	Automated lumen contours using local transmittance-based enhancement	8100 images (30 pullbacks, 270 images per pullback)	Polar transform, transmissivity-based mapping	Region-based level set active contour method	BR DICE: 0.78 ± 0.20	Expert annotation
Macedo et al. [62]	Automated lumen segmentation by morphological operations in plaque and bifurcation regions.	1328 images (nine pullbacks, 141 BR, 1188 NBR)	Polar transform, Bilateral filtering, Otsu thresholding, Erosion/dilation	Sobel edge detection, Distance transform based automatic contour correction	NBR MADA: 0.19 ± 0.13 mm^2^ NBR DICE: 0.97 ± 0.02 BR MADA: 0.52 ± 0.81 mm^2^ BR DICE: 0.91 ± 0.09	Manual segmentation
Miyagawa et al. [77]	Automated detection and outline of bifurcation regions	2460 images (Nine patients, 157 BR, 1204 NBR, 1099 DA)	Global thresholding, closing, Hough transform	Four CNNs, three with transfer learning from lumen detection	ACC: 98.00 ± 1.00% SPE: 98.00 ± 1.00% AUC: 0.99 ± 0.00	Expert annotation
Pociask et al. [66]	Automated lumen segmentation	667 images	Polar transform, Gaussian & Savitzky–Golay filtering, opening/closing	Linear interpolation	Relative difference in lumen area: 1.12% (1.55–0.68%)	Manual segmentation
Roy et al. [69]	Random walks automatic segmentation of the lumen	Patients: six in vivo, 15 in vitro. 150–300 frames per patient	Polar transform,	Random walks based on edge weights and backscattering tracking	CK: 0.98 ± 0.01 KL: 5.17 ± 2.39 BHAT: 0.56 ± 0.28	Expert annotation
Tang et al. [87]	Automated lumen extraction using N-Net CNN	20,000 images (400 for training from manual annotation)		N-Net CNN with cross entropy loss function	ACC: 98.00 ± 0.00% DICE: 0.93 ± 0.00 JS: 0.88 ± 0.00 SPE: 99.00 ± 0.00%	Expert annotation of 400 images
Yang et al. [84]	Automated lumen extraction in abnormal lumen geometries	14,207 images (54 patients)	Polar transform, Gaussian filtering	Active contour model, Gray-level co-occurrence matrix, SVM, AdaBoost, J48, RF, NB, Bagging	DICE: 0.98 ± 0.01 JS: 0.95 ± 0.02 MADA: 0.27 ± 0.19 mm^2^ ASSD: 0.03 ± 0.01 mm RMSD: 0.04 ± 0.01 mm ACC: 99.00 ± 1.00%	Expert annotation on 1541 images
Yong et al. [85]	Automated lumen extraction using linear regression CNN	19,027 images (64 pullbacks, 28 patients)	Polar transform,	Linear regression CNN	Location accuracy: 22 µm DICE 0.99 JS: 0.97	Expert annotation on 19 pullbacks (5685 images)
Zhao et al. [61]	Automated lumen extraction using morphological operations	268 images	Polar transform, Median filtering, Otsu binarization, closing/opening		DICE: 0.99 JS: 0.99 ACC: 99.00% HD: 0.01 mm	Expert annotation
Zhu et al. [59]	Automated lumen segmentation to overcome blood artefacts	216 images with blood artefacts (from 1436 images, 6 patients)	Polar transform, Gaussian filtering, adaptive block binarization, erosion/area opening	Connected A-line region filtering with bicubic interpolation and quadratic regression smoothing	DICE: 0.95 JS: 0.90 ACC: 98.00%	Morphological only, dynamic programming, manual segmentation

**Table A2:** Classified articles investigating automated artery layer segmentation. ACC—Accuracy. APe—Adventitia-peri-adventitial tissue border error. CNN—Convolutional neural network. DICE—Dice loss coefficient. IMe—Intima-media border error. IVUS—Intravascular ultrasound. JS—Jaccard similarity index. MADA—Mean absolute difference in area. MAe—Media-adventitia border error. OCT—Optical coherence tomography. R^2^—Coefficient of determination. RF—Random Forest. SEN—Sensitivity. SPE—Specificity. SVM—Support vector machine. * Results shown for the outer wall segmentation.

First Author [Ref]	Aim	Dataset	Morphological Operations	Feature Detection/Classification	Outcome	Comparison
Abdolmanafi et al. [101]	Automated intima and media classification in pediatric patients	4800 regions of interest (26 patients)		CNN (AlexNet), RF, SVM	CNN ACC: 97.00 ± 4.00% RF ACC: 96.00 ± 6.00% SVM ACC: 90.00 ± 10.00%	Manual segmentation
Chen et al. [102]	Automated wall morphology change analyses in heart transplant patients	43,873 images (100 pullbacks, 50 patients)		Caffe framework, LOGISMOS, Sobel edge detector	R^2^: 0.96 Intima error: 4.98 ± 31.24 µm Media error: 5.38 ± 28.54 µm	Expert annotation
Haft-Javaherian et al. [110]	Automated lumen, intima and media classification in polarization-sensitive OCT	984 images (57 patients)		CNN based on U-Net and deep residual learning model, combination of five loss functions	DICE *: 0.99 ACC *: 99.30% SEN *: 99.50% SPE *: 99.00%	Expert annotation and traditional OCT.
Olender et al. [113]	Automated delineation of outer elastic membrane using mechanical approach	724 images (seven patients)	Contrast enhancement, image compensation, median filtering	Sobel-Feldman edge detection, anisotropic linear elastic mesh force balance	MADA: 0.93 mm^2^ (±0.84) DICE: 0.91 JS: 0.84 SEN: 90.79% SPE: 99.00%	Expert annotation and IVUS
Pazdernik et al. [103]	Automated wall morphology change analyses in heart transplant patients	50 patients (~25,000 co-registered images)		LOGISMOS	R^2^: 0.99 Intima error: 0.4 ± 27.1 µm Media error: 8.1 ± 12.2 µm	Expert annotation
Zahnd et al. [100]	Automatically segment three layers of healthy coronary artery wall	40 patients (400 classified images, 140 training, 260 validation)	Erosion, dilation	AdaBoost, front propagation scheme with cumulative cost function, Boruta algorithm (RF based)	DICE: 0.93 ACC: 91.00% SEN: 92.00% SPE: 100.00% IMe: 29 ± 46 µm MAe: 30 ± 50 µm APe: 50 ± 64 µm	Expert annotation

**Table A3:** Classified articles investigating automated plaque classification and segmentation. ACC—Accuracy. ADAM—Gradient based adaptive optimization. AFPDEFCM—Fourth-order PDE-based fuzzy c-means. ANN—Artificial neural network. AP—Average precision. AUC—Area under the curve. CNN—Convolutional neural network. CRF—Conditional random field. DA—Data augmentation. DB—Dual binary classifier. DICE—Dice loss coefficient. EEL—External elastic lamina. F1—F1-score. FC—Fibrocalcific plaque. FCM—Partial differential equation-based fuzzy c-means. FCN—Fully convolutional network. FRSCGMM—Fast and robust spatially constrained Gaussian mixture model. GMM—Gaussian mixture model. GMM-SMSI—GMM with spatial pixel saliency map. HEM—Heard example mining. HER—Healed erosion/rupture. MCR—Misclassification ratio. MIoU—Mean intersection over union. FIoU—Frequency weighted intersection over union. mRMR—Minimal-redundancy-maximal relevance. PB—Plaque burden. PIT—Pathological intimal thickening. PRE—Precision. PRI—Probabilistic Rand Index. REC—Recall. RF—Random Forest. SEN—Sensitivity. SMM—Student’s-t mixture model. SPE—Specificity. SVM—Support vector machine. TCFA—Thin-cap fibroatheroma. VH-IVUS—Virtual histology intravascular ultrasound. VOI—Volume of interest. * Overall classification accuracy for fibrous, lipid and background tissue. ** Mean values for presented algorithm, see text for other comparison metrics. ^ Results for the final contraction plus expansion CNN. ^^ Results for overall pathological tissue detection.

First Author [Ref]	Aim	Dataset	Morphological Operations	Feature Detection/Classification	Outcome	Comparison
Abdolmanafi et al. [132]	Tissue characterization in Kawasaki disease	8910 images (33 pullbacks)	Polar transform	RF (AlexNet, VGG-19 & Inception-V3) & majority voting	ACC ^^: 99.00 ± 1.00% SEN: 98.00 ± 2.00% SPE: 100.00 ± 0.00%	Expert annotation
Abdolmanafi et al. [133]	Tissue characterization in Kawasaki disease	5040 images (45 pullbacks)	Polar transform	FCN, RF (VGG-19)	ACC ^^: 96.00 ± 4.00% SPE: 95.00 ± 5.00% SEN: 97.00 ± 3.00% F1: 0.96 ± 0.04	Expert annotation
Abdolmanafi et al. [134]	Automatic plaque tissue classification	41 pullbacks (~200 images per pullback)		FCN (ResNet), ADAM optimizer	ACC: 93.00 ± 10.00% SEN: 90.00 13.00% SPE: 95.00 ± 5.00% F1: 0.84 ± 0.18	Manual segmentation
Avital et al. [168]	Deep learning-based calcification classification	8000 images (540 frames for training)		U-Net	ACC: 99.03 ± 9.00% DICE: 0.71 ± 0.26	Manual segmentation
Cheimariotis et al. [161]	Four-way plaque type classification	183 images (33 patients)	Polar transform, Median filtering, Gaussian filtering, opening, Otsu binarization, low-pass filtering (ARC-OCT)	CNN (AlexNet), ADAM optimizer with attenuation coefficient	A-line transformed ACC: 83.47% Plaque: ACC: 74.73% SEN: 87.78% SPE: 61.45%	Manual segmentation
Gerbaud et al. [151]	Plaque burden measurement with enhancement algorithm	42 patients (96 pullbacks) 200 IVUS-OCT matched images	Adaptive attenuation compensation, frame averaging		Mean difference. EEL: 0.27 ± 3.31 mm^2^ PB: −0.5 ± 7.0%	Expert annotation and IVUS
Gessert et al. [130]	Plaque detection and segmentation with multi-path architecture	4000 images (49 patients)	Polar & cartesian	CNN (ResNet50-V2 & DenseNet-121)	ACC: 91.70% SEN: 90.90% SPE: 92.40% F1: 0.91	Expert annotation
Gharaibeh et al. [170]	Classification and segmentation of lumen and calcification	2640 images (34 pullbacks)	Polar transform, log-transform, Gaussian filtering	CNN (SegNet) & CRF	Calcific: DICE: 0.76 ± 0.03 SEN: 85.00 ± 4.00% Lumen: DICE: 0.98 ± 0.01 SEN: 99.00 ± 1.00%	Manual segmentation
He et al. [167]	Automatic classification of calcification	4860 images (18 pullbacks)	Polar transform	CNN (ResNet-3D & 2D), cross-entropy loss, ADAM optimizer	PRE: 96.90 ± 1.30% REC: 97.70 ± 3.40% F1: 96.10 ± 3.40%	Manual segmentation
Huang et al. [136]	Fibrous, calcific and lipidic tissue classification	28 images (11 patients]	Polar transform, Otsu thresholding,	SVM (RF feature selection)	ACC: 83.00% Fibrous ACC: 89.00% Lipidic ACC: 86.50% Calcific ACC: 79.30%	Manual segmentation
Isidori et al. [152]	Automated lipid core burden index assessment	Training: 23 patients. Testing: 40 patients,		CNN	SEN: 90.50% SPE: 84.20%	Expert annotation and NIRS-IVUS
Kolluru et al. [155]	CNN classification of plaque types (fibro-calcific and fibro-lipidic)	4469 images (48 pullbacks)	Log transform, Gaussian filtering	CNN and ANN	ACC: 77.7% ± 4.1% for fibro-calcific, 86.5% ± 2.3% for fibro-lipid and 85.3% ± 2.5% for others	Expert annotation and ANN
Kolluru et al. [172]	Reduce number of training images needed for deep learning	3741 images (60 VOIs from 41 pullbacks)	Log transform, Gaussian filtering	U-Net, Image subset selection through deep-feature clustering and k-medoids algorithm	Clustering outperforms equal spacing methods for sparse annotations (F1: 0.63 vs. 0.52, AP: 66% vs. 50%)	Expert annotation
Lee et al. [156]	Hybrid learning approach to classify fibro-lipidic and fibro-calcific tissue	6556 images	Polar transform, Gaussian filtering	CNN (ADAM optimizer) & RF with hybrid learning approach, CRF & dynamic programming	Fibro-lipidic: SEN: 84.80 ± 8.20% SPE: 97.80 ± 1.60% F1: 0.89 ± 0.04 Fibro-calcific: SEN: 91.20 ± 6.40% SPE: 96.20 ± 1.60% F1: 0.72 ± 0.07	Manual segmentation, pre & post noise cleaning and active learning
Lee et al. [157]	Automatic lipid/calcium characterization comparison	4892 images (57 pullbacks, 55 patients)	Polar transform, non-local mean filtering	CNN (SegNet VGG16), Deeplab 3+, dynamic programming		Manual segmentation, pixel-wise vs. A-line
Lee et al. [169]	Fully automated 3D calcium segmentation and reconstruction	8231 images (68 patients) 4320 ex vivo images (four cadavers)	Polar transform, Gaussian filtering, opening & closing	3D CNN & SegNet with Tversky loss function, CRF & dynamic programming	SEN: 97.70% SPE: 87.70% F1: 0.92	Manual segmentation, one-step approach
Li et al. [135]	Segmentation of vulnerable plaque regions	2000 images (50% vulnerable plaque)	Polar transform	Deep Residual U-Net (ResNet101) & combined cross-entropy and dice loss	ACC: 93.31% MIoU: 0.85 FIoU: 0.86 PRE: 94.33% REC: 91.35%	Manual segmentation, prototype U-Net; VGG16, ResNet50, ResNet101
Liu et al. [144]	Automated fibrous plaque detection	1000 images	Polar & Hough transform	CNN (VGG16)	ACC ^: 94.12% REC: 94.12%	Expert annotation, SSD, YOLO-V3
Liu et al. [150]	Vulnerable plaque detection	2000 training images, 300 testing images, data augmentation	Polar transform, erosion/dilation, de-noising	Deep CNN (Adaboost, YOLO, SSD, Faster R-CNN)	PRE: 88.84% REC: 95.02%	Manual segmentation
Liu et al. [162]	Classification of six tissue types: mixed, calcification, fibrous, lipid-rich, macrophages, necrotic core	135 images (ex vivo)	Polar transform, median filtering	Attenuation, backscatter, intensity	Attenuation and backscatter can differentiate six tissue types	Expert annotation & histology
Prabhu et al. [115]	Detection of fibro-lipidic and fibro-calcific A-lines	6556 in vivo images (49 pullbacks), 440 ex vivo images (10 pullbacks)	Polar transform, texture features from Leung–Malik filter bank	RF, SVM, DB, mRMR, binary Wilcoxon & CRF	ACC: 81.58% Fibro-lipidic: SEN: 94.48% SPE: 87.32% Fibro-calcific: SEN: 74.82% SPE: 95.28%	Expert annotation
Rico-Jimenez et al. [129]	Automated tissue characterization with A-line features	513 images	Polar transform, entropy & frost filter	Linear Discriminant Analysis	ACC: 88.20%	Manual segmentation
Rico-Jimenez et al. [153]	Macrophage infiltration detection	28 ex vivo coronary segments	Normalized-intensity standard deviation ratio		ACC: 87.45% SEN: 85.57% SPE: 88.03%	Manual segmentation and histological evaluation
Shibutani et al. [154]	Automated plaque characterization in ex vivo sections	1103 histological cross sections (45 autopsied hearts)		CNN (ResNet50), scene parsing network (PSPNet)	FC AUC: 0.91 PIT AUC: 0.85 TCFA AUC: 0.86 HER AUC: 0.86	Expert annotation and histological evaluation
Wang et al. [128]	Fibrotic plaque area segmentation	20 images (nine patients)	Adaptive diffusivity	Log-likelihood function of Gaussian mixture model (GMM)	MCR **: 0.65 ± 0.66 PRI: 0.99 ± 0.01	Manual segmentation, GMM, FCM, SMM, FRSCGMM, AFPDEFCM, GMM-SMSI
Yang et al. [127]	Automatic classification of plaque (fibrous, calcific and lipid-rich)	1700 images (20 pullbacks, nine patients)	Mean filtering, graph-cut method	SVM (C-SVC) with HEM training, K-means, radial basis function	ACC: 96.80 ± 0.02%	Manual segmentation
Zhang et al. [120]	Automated fibrous cap thickness quantification and plaque classification	18 images (two patients, 1008 images after DA)		CNN (U-Net), CNN (FC-DenseNet), SVM	U-Net ACC *: 95.40% FC-DenseNet ACC: 91.14% SVM ACC: 81.84%	Manual segmentation guided by VH-IVUS
Zhang et al. [126]	Comparison of automated lipid, fibrous and background tissue segmentation	77 images (five patients)		CNN (U-Net based architecture) and SVM Focal loss function, local binary patterns, gray level co-occurrence matrices	CNN ACC *: 94.29% SVM ACC: 69.46%	Manual segmentation guided by VH-IVUS

**Table A4:** Classified articles investigating automated stent segmentation. 3D—Three-dimensional. ADAM—Gradient based adaptive optimization. ANN—Artificial neural network. AP—Average precision. ASSD—Average symmetric surface distance. AUC—Area under the curve. CCC—Concordance-correlation-coefficient. CFD—Computational fluid dynamics. CT—Computed Tomography DA—Data augmentation. DICE—Dice loss coefficient. F1—F1-score. FPR—False positive ratio. JS—Jaccard similarity index. MADA—Mean average difference in area. OCT—Optical coherence tomography. PPV—Positive predictive value. PRE—Precision. R^2^—Coefficient of determination. REC—Recall. SEN—Sensitivity. SPE—Specificity. SVM—Support vector machine. TPR—True positive ratio. * Results for the best outcome are shown in the Table, please refer to the article for detailed inter/intra-observer variability and method comparisons.

First Author [Ref]	Aim	Dataset	Morphological Operations	Feature Detection/Classification	Outcome	Comparison
Bologna et al. [64]	Automated lumen contour and stent strut selection for 3D reconstruction	1150 images (23 pullbacks)	Thresholding, opening, closing, nonlinear filtering	Sobel edge detection	Lumen: SPE: 97.00% SEN: 99.00% Stent: SPE: 63.00% SEN: 83.00%	Manual segmentation
Cao et al. [176]	Automatic stent segmentation and malapposition evaluation	4065 images (12,550 struts, 15 pullbacks)		Cascade AdaBoost classifier, dynamic programming	DICE: 0.81 TPR: 90.50% FPR: 12.10% F1: 0.90	Expert annotation
Chiastra et al. [187]	Stent strut and lumen contour detection through OCT and micro-CT	Eight stented bifurcation phantom arteries (in vitro), four in vivo patients	Polar transform, opening, thresholding	Sobel edge detection	Stent *: DICE: 0.93 ± 0.06 JS: 0.87 ± 0.10 SPE: 94.75 ± 7.60% SEN: 90.87 ± 9.44%	Manual segmentation
Elliot et al. [190]	Automated 3D stent reconstruction through OCT and micro-CT	2156 images, four stented phantom arteries (in vitro)	Polar transform	A-line intensity profile, peak intensity, number of peaks	ASSD: 184 ± 96 µm	Manual segmentation
Jiang et al. [178]	Automatic segmentation of metallic stent struts	165 images, 1200 post DA on (10 pullbacks)		YOLOv3 (binary cross-entropy loss) and region-based fully-convolutional network (R-FCN), Darknet53	YOLOv3 vs. R-FCN PRE: 97.20% vs. 99.80% REC: 96.50% vs. 96.20% AP: 96.00% vs. 96.20%	Manual segmentation and between two classifiers
Junedh et al. [179]	Automation of polymeric stent strut segmentation	1140 images (15 patients)	Polar transform, bilateral filter	K-means	R^2^: 0.88 PPV: 93.00% TPR: 90.00%	Expert annotation
Lau et al. [180]	Segmentation of metallic and bioresorbable vascular scaffolds	51 pullbacks (27 patients), 13,890 training images, 3909 test images		U-Net with combined MobileNetV2 and DenseNet121	DICE *: 0.86 PRE *: 92.00% REC *: 92.00%	Manual segmentation
Lu et al. [182]	Automatic classification of covered/uncovered stents	7125 images (39,000 covered struts, 16,500 uncovered struts, 80 pullbacks)	Polar transform	SVM (LIBSVM), bagged decision trees classifier, pixel patch method, mesh growing, active learning relabeling	SPE: 94.00 ± 3.00% SEN: 90.00 ± 4.00% AUC: 0.97	Expert annotation
Lu et al. [184]	Development of automated OCT image visualization and analysis toolkit for stents (OCTivat-stent)	(292 pullbacks)	Polar transform	SVM (LIBSVM), bagged decision trees classifier, pixel patch method, mesh growing, active learning relabeling	Lumen CCC: 0.99 Stent CCC: 0.97	Expert annotation
Migliori et al. [189]	Framework for automated stent segmentation and lumen reconstruction for CFD simulation	540 images, 0ne phantom (in vitro)	Polar transform, intensity/area thresholding	Fuzzy logic, Sobel edge detection and linear interpolation	Stent *: DICE: 0.87 ± 0.13 JS: 0.78 ± 0.18% SPE: 77.8 ± 28.20% SEN: 91.7 ± 13.20%	Manual segmentation of 95 images
Nam et al. [174]	Automatic stent apposition and neointimal coverage analysis	5420 images (20 pullbacks)	Polar transform, Gaussian smoothing	ANN, image gradient and intensity	PPV: 95.60% TPR: 92.90%	Manual segmentation on 800 images
O’Brien et al. [186]	Enhanced stent and lumen 3D reconstruction for CFD simulation	Four swine pullbacks		Decision tree, ramp edge detection	Lumen (62 frames) MADA: 0.42 ± 0.13 mm^2^ Stent (57 frames) MADA: 0.20 ± 0.17 mm^2^	Manual segmentation
Wu et al. [175]	Automated stent strut detection in multiple stent designs	Training: 10,417 images (60 pullbacks) Testing: 21,363 images (170 pullbacks)	Polar transform, Manual training mask	U-Net based deep convolutional model (ADAM optimizer, binary cross-entropy and Tversky loss functions)	DICE: 0.91 ± 0.04 JS: 0.84 ± 0.06 PRE: 94.30 ± 3.60% REC: 94.00 ± 3.90% F1: 0.94 ± 0.04	Expert annotation and QIvus v3.1 (Medis Medical Imaging System BV, Leiden, The Netherlands)

## References

[1] Virani S.S., Alonso A., Aparicio H.J., Benjamin E.J., Bittencourt M.S., Callaway C.W., Carson A.P., Chamberlain A.M., Cheng S., Delling F.N. (2021). Heart disease and stroke statistics—2021 update: A report from the American Heart Association. Circulation.

[2] Gheorghe A., Griffiths U., Murphy A., Legido-Quigley H., Lamptey P., Perel P. (2018). The economic burden of cardiovascular disease and hypertension in low-and middle-income countries: A systematic review. BMC Public Health.

[3] Jernberg T., Hasvold P., Henriksson M., Hjelm H., Thuresson M., Janzon M. (2015). Cardiovascular risk in post-myocardial infarction patients: Nationwide real world data demonstrate the importance of a long-term perspective. Eur. Heart J..

[4] Baumann A.A.W., Mishra A., Worthley M.I., Nelson A.J., Psaltis P.J. (2020). Management of multivessel coronary artery disease in patients with non-ST-elevation myocardial infarction: A complex path to precision medicine. Ther. Adv. Chronic Dis..

[5] Libby P., Ridker P.M., Hansson G.K. (2011). Progress and challenges in translating the biology of atherosclerosis. Nature.

[6] Kim W.Y., Danias P.G., Stuber M., Flamm S.D., Plein S., Nagel E., Langerak S.E., Weber O.M., Pedersen E.M., Schmidt M. (2001). Coronary magnetic resonance angiography for the detection of coronary stenoses. N. Engl. J. Med..

[7] Narula J., Nakano M., Virmani R., Kolodgie F.D., Petersen R., Newcomb R., Malik S., Fuster V., Finn A.V. (2013). Histopathologic characteristics of atherosclerotic coronary disease and implications of the findings for the invasive and noninvasive detection of vulnerable plaques. J. Am. Coll. Cardiol..

[8] Xie Z., Tian J., Ma L., Du H., Dong N., Hou J., He J., Dai J., Liu X., Pan H. (2015). Comparison of optical coherence tomography and intravascular ultrasound for evaluation of coronary lipid-rich atherosclerotic plaque progression and regression. Eur. Heart J. Cardiovasc. Imaging.

[9] Tearney G.J., Waxman S., Shishkov M., Vakoc B.J., Suter M.J., Freilich M.I., Desjardins A.E., Oh W.-Y., Bartlett L.A., Rosenberg M. (2008). Three-dimensional coronary artery microscopy by intracoronary optical frequency domain imaging. JACC Cardiovasc. Imaging.

[10] Prati F., Romagnoli E., Gatto L., La Manna A., Burzotta F., Ozaki Y., Marco V., Boi A., Fineschi M., Fabbiocchi F. (2019). Relationship between coronary plaque morphology of the left anterior descending artery and 12 months clinical outcome: The CLIMA study. Eur. Heart J..

[11] Montarello N.J., Nelson A.J., Verjans J., Nicholls S.J., Psaltis P.J. (2020). The role of intracoronary imaging in translational research. Cardiovasc. Diagn. Ther..

[12] Carpenter H.J., Gholipour A., Ghayesh M.H., Zander A.C., Psaltis P.J. (2020). A review on the biomechanics of coronary arteries. Int. J. Eng. Sci..

[13] Shishikura D., Sidharta S.L., Honda S., Takata K., Kim S.W., Andrews J., Montarello N., Delacroix S., Baillie T., Worthley M.I. (2018). The relationship between segmental wall shear stress and lipid core plaque derived from near-infrared spectroscopy. Atherosclerosis.

[14] Giannoglou G.D., Soulis J.V., Farmakis T.M., Farmakis D.M., Louridas G.E. (2002). Haemodynamic factors and the important role of local low static pressure in coronary wall thickening. Int. J. Cardiol..

[15] Bourantas Christos V., Räber L., Sakellarios A., Ueki Y., Zanchin T., Koskinas Konstantinos C., Yamaji K., Taniwaki M., Heg D., Radu Maria D. (2020). Utility of multimodality intravascular imaging and the local hemodynamic forces to predict atherosclerotic disease progression. JACC Cardiovasc. Imaging.

[16] Soulis J.V., Fytanidis D.K., Papaioannou V.C., Giannoglou G.D. (2010). Wall shear stress on LDL accumulation in human RCAs. Med. Eng. Phys..

[17] Bourantas Christos V., Zanchin T., Torii R., Serruys Patrick W., Karagiannis A., Ramasamy A., Safi H., Coskun Ahmet U., Koning G., Onuma Y. (2020). Shear stress estimated by quantitative coronary angiography predicts plaques prone to progress and cause events. JACC Cardiovasc. Imaging.

[18] Stone P.H., Maehara A., Coskun A.U., Maynard C.C., Zaromytidou M., Siasos G., Andreou I., Fotiadis D., Stefanou K., Papafaklis M. (2018). Role of low endothelial shear stress and plaque characteristics in the prediction of nonculprit major adverse cardiac events: The PROSPECT study. JACC Cardiovasc. Imaging.

[19] Gholipour A., Ghayesh M.H., Zander A., Mahajan R. (2018). Three-dimensional biomechanics of coronary arteries. Int. J. Eng. Sci..

[20] Pei X., Wu B., Li Z.-Y. (2013). Fatigue crack propagation analysis of plaque rupture. J. Biomech. Eng..

[21] Cardoso L., Weinbaum S. (2014). Changing views of the biomechanics of vulnerable plaque rupture: A review. Ann. Biomed. Eng..

[22] Wang L., Wu Z., Yang C., Zheng J., Bach R., Muccigrosso D., Billiar K., Maehara A., Mintz G.S., Tang D. (2015). IVUS-based FSI models for human coronary plaque progression study: Components, correlation and predictive analysis. Ann. Biomed. Eng..

[23] Carpenter H., Gholipour A., Ghayesh M., Zander A.C., Psaltis P. (2021). In vivo based fluid-structure interaction biomechanics of the left anterior descending coronary artery. J. Biomech. Eng..

[24] Wang Q., Tang D., Wang L., Meahara A., Molony D., Samady H., Zheng J., Mintz G.S., Stone G.W., Giddens D.P. (2021). Multi-patient study for coronary vulnerable plaque model comparisons: 2D/3D and fluid–structure interaction simulations. Biomech. Model. Mechanobiol..

[25] Tang D., Yang C., Kobayashi S., Zheng J., Woodard P.K., Teng Z., Billiar K., Bach R., Ku D.N. (2009). 3D MRI-based anisotropic FSI models with cyclic bending for human coronary atherosclerotic plaque mechanical analysis. J. Biomech. Eng..

[26] Costopoulos C., Brown A.J., Teng Z., Hoole S.P., West N.E.J., Samady H., Bennett M.R. (2016). Intravascular ultrasound and optical coherence tomography imaging of coronary atherosclerosis. Int. J. Cardiovasc. Imaging.

[27] Fujimoto J.G. (2003). Optical coherence tomography for ultrahigh resolution in vivo imaging. Nat. Biotechnol..

[28] Bezerra Hiram G., Costa Marco A., Guagliumi G., Rollins Andrew M., Simon Daniel I. (2009). Intracoronary optical coherence tomography: A comprehensive review. JACC Cardiovasc. Interv..

[29] Prati F., Regar E., Mintz G.S., Arbustini E., Di Mario C., Jang I.-K., Akasaka T., Costa M., Guagliumi G., Grube E. (2010). Expert review document on methodology, terminology, and clinical applications of optical coherence tomography: Physical principles, methodology of image acquisition, and clinical application for assessment of coronary arteries and atherosclerosis. Eur. Heart J..

[30] Jang I.-K., Bouma B.E., Kang D.-H., Park S.-J., Park S.-W., Seung K.-B., Choi K.-B., Shishkov M., Schlendorf K., Pomerantsev E. (2002). Visualization of coronary atherosclerotic plaques in patients using optical coherence tomography: Comparison with intravascular ultrasound. J. Am. Coll. Cardiol..

[31] Kim S.-J., Lee H., Kato K., Yonetsu T., Xing L., Zhang S., Jang I.-K. (2012). Reproducibility of in vivo measurements for fibrous cap thickness and lipid arc by OCT. JACC Cardiovasc. Imaging.

[32] Koskinas K.C., Ughi G.J., Windecker S., Tearney G.J., Räber L. (2016). Intracoronary imaging of coronary atherosclerosis: Validation for diagnosis, prognosis and treatment. Eur. Heart J..

[33] Nakajima A., Araki M., Minami Y., Soeda T., Yonetsu T., McNulty I., Lee H., Nakamura S., Jang I.-K. (2021). Layered plaque characteristics and layer burden in acute coronary syndromes. Am. J. Cardiol..

[34] Araki M., Yonetsu T., Kurihara O., Nakajima A., Lee H., Soeda T., Minami Y., McNulty I., Uemura S., Kakuta T. (2021). Predictors of rapid plaque progression: An optical coherence tomography study. JACC Cardiovasc. Imaging.

[35] Araki M., Park S.-J., Dauerman H.L., Uemura S., Kim J.-S., Di Mario C., Johnson T.W., Guagliumi G., Kastrati A., Joner M. (2022). Optical coherence tomography in coronary atherosclerosis assessment and intervention. Nat. Rev. Cardiol..

[36] Montarello N.J., Singh K., Sinhal A., Wong D.T.L., Alcock R., Rajendran S., Dautov R., Barlis P., Patel S., Nidorf S.M. (2021). Assessing the impact of colchicine on coronary plaque phenotype after myocardial infarction with optical coherence tomography: Rationale and design of the COCOMO-ACS study. Cardiovasc. Drugs Ther..

[37] Nicholls S.J., Nissen S.E., Prati F., Windecker S., Kataoka Y., Puri R., Hucko T., Kassahun H., Liao J., Somaratne R. (2021). Assessing the impact of PCSK9 inhibition on coronary plaque phenotype with optical coherence tomography: Rationale and design of the randomized, placebo-controlled HUYGENS study. Cardiovasc. Diagn. Ther..

[38] Habara M., Nasu K., Terashima M., Ko E., Yokota D., Ito T., Kurita T., Teramoto T., Kimura M., Kinoshita Y. (2014). Impact on optical coherence tomographic coronary findings of fluvastatin alone versus fluvastatin+ ezetimibe. Am. J. Cardiol..

[39] Komukai K., Kubo T., Kitabata H., Matsuo Y., Ozaki Y., Takarada S., Okumoto Y., Shiono Y., Orii M., Shimamura K. (2014). Effect of atorvastatin therapy on fibrous cap thickness in coronary atherosclerotic plaque as assessed by optical coherence tomography: The EASY-FIT study. J. Am. Coll. Cardiol..

[40] Gholipour A., Ghayesh M.H., Zander A.C., Psaltis P.J. (2020). In vivo based biomechanics of right and left coronary arteries. Int. J. Eng. Sci..

[41] Toutouzas K., Chatzizisis Y.S., Riga M., Giannopoulos A., Antoniadis A.P., Tu S., Fujino Y., Mitsouras D., Doulaverakis C., Tsampoulatidis I. (2015). Accurate and reproducible reconstruction of coronary arteries and endothelial shear stress calculation using 3D OCT: Comparative study to 3D IVUS and 3D QCA. Atherosclerosis.

[42] Migliori S., Chiastra C., Bologna M., Montin E., Dubini G., Genuardi L., Aurigemma C., Mainardi L., Burzotta F., Migliavacca F. (2020). Application of an OCT-based 3D reconstruction framework to the hemodynamic assessment of an ulcerated coronary artery plaque. Med. Eng. Phys..

[43] Wang L., He L., Jia H., Lv R., Guo X., Yang C., Giddens D.P., Samady H., Maehara A., Mintz G. (2021). Optical coherence tomography-based patient-specific residual multi-thrombus coronary plaque models with fluid-structure interaction for better treatment decisions: A biomechanical modeling case study. J. Biomech. Eng..

[44] Carpenter H.J., Ghayesh M.H., Zander A.C., Ottaway J.L., Di Giovanni G., Nicholls S.J., Psaltis P.J. (2022). Optical coherence tomography based biomechanical fluid-structure interaction analysis of coronary atherosclerosis progression. J. Vis. Exp. JoVE.

[45] Tearney G.J., Regar E., Akasaka T., Adriaenssens T., Barlis P., Bezerra H.G., Bouma B., Bruining N., Cho J.-M., Chowdhary S. (2012). Consensus standards for acquisition, measurement, and reporting of intravascular optical coherence tomography studies: A report from the International Working Group for Intravascular Optical Coherence Tomography Standardization and Validation. J. Am. Coll. Cardiol..

[46] LeCun Y., Bengio Y., Hinton G. (2015). Deep learning. Nature.

[47] De Boer P.-T., Kroese D.P., Mannor S., Rubinstein R.Y. (2005). A tutorial on the cross-entropy method. Ann. Oper. Res..

[48] Sudre C.H., Li W., Vercauteren T., Ourselin S., Jorge Cardoso M., Cardoso M.J., Arbel T., Carneiro G., Syeda-Mahmood T., Tavares J.M.R.S. (2017). Generalised Dice Overlap as a Deep Learning Loss Function for Highly Unbalanced Segmentations. Proceedings of the Deep Learning in Medical Image Analysis and Multimodal Learning for Clinical Decision Support.

[49] Salehi S.S.M., Erdogmus D., Gholipour A., Wang Q., Shi Y., Suk H.-I., Suzuki K. (2017). Tversky Loss Function for Image Segmentation Using 3D Fully Convolutional Deep Networks. Proceedings of the Machine Learning in Medical Imaging.

[50] Sony S., Dunphy K., Sadhu A., Capretz M. (2021). A systematic review of convolutional neural network-based structural condition assessment techniques. Eng. Struct..

[51] Hassoun M.H. (1995). Fundamentals of Artificial Neural Networks.

[52] Chen L.-C., Papandreou G., Kokkinos I., Murphy K., Yuille A.L. (2014). Semantic image segmentation with deep convolutional nets and fully connected crfs. arXiv.

[53] Ronneberger O., Fischer P., Brox T., Navab N., Hornegger J., Wells W.M., Frangi A.F. (2015). U-Net: Convolutional Networks for Biomedical Image Segmentation. Proceedings of the Medical Image Computing and Computer-Assisted Intervention—MICCAI 2015.

[54] Litjens G., Ciompi F., Wolterink Jelmer M., de Vos Bob D., Leiner T., Teuwen J., Išgum I. (2019). State-of-the-art deep learning in cardiovascular image analysis. JACC Cardiovasc. Imaging.

[55] Tajbakhsh N., Jeyaseelan L., Li Q., Chiang J.N., Wu Z., Ding X. (2020). Embracing imperfect datasets: A review of deep learning solutions for medical image segmentation. Med. Image Anal..

[56] Gudigar A., Nayak S., Samanth J., Raghavendra U., AJ A., Barua P.D., Hasan M.N., Ciaccio E.J., Tan R.-S., Rajendra Acharya U. (2021). Recent trends in artificial intelligence-assisted coronary atherosclerotic plaque characterization. Int. J. Environ. Res. Public Health.

[57] Boi A., Jamthikar A.D., Saba L., Gupta D., Sharma A., Loi B., Laird J.R., Khanna N.N., Suri J.S. (2018). A survey on coronary atherosclerotic plaque tissue characterization in intravascular optical coherence tomography. Curr. Atheroscler. Rep..

[58] Johnson Kipp W., Torres Soto J., Glicksberg Benjamin S., Shameer K., Miotto R., Ali M., Ashley E., Dudley Joel T. (2018). Artificial intelligence in cardiology. J. Am. Coll. Cardiol..

[59] Zhu F., Ding Z., Tao K., Li Q., Kuang H., Tian F., Zhou S., Hua P., Hu J., Shang M. (2021). Automatic lumen segmentation using uniqueness of vascular connected region for intravascular optical coherence tomography. J. Biophotonics.

[60] Otsu N. (1979). A threshold selection method from gray-level histograms. IEEE Trans. Syst. Man Cybern..

[61] Zhao H., He B., Ding Z., Tao K., Lai T., Kuang H., Liu R., Zhang X., Zheng Y., Zheng J. (2019). Automatic lumen segmentation in intravascular optical coherence tomography using morphological features. IEEE Access.

[62] Macedo M.M.G.D., Takimura C.K., Lemos P.A., Gutierrez M.A. (2016). A robust fully automatic lumen segmentation method for in vivo intracoronary optical coherence tomography. Res. Biomed. Eng..

[63] Cheimariotis G.-A., Chatzizisis Y.S., Koutkias V.G., Toutouzas K., Giannopoulos A., Riga M., Chouvarda I., Antoniadis A.P., Doulaverakis C., Tsamboulatidis I. (2017). ARCOCT: Automatic detection of lumen border in intravascular OCT images. Comput. Methods Programs Biomed..

[64] Bologna M., Migliori S., Montin E., Rampat R., Dubini G., Migliavacca F., Mainardi L., Chiastra C. (2019). Automatic segmentation of optical coherence tomography pullbacks of coronary arteries treated with bioresorbable vascular scaffolds: Application to hemodynamics modeling. PLoS ONE.

[65] Akbar A., Khwaja T.S., Javaid A., Kim J.-S., Ha J. (2019). Automated accurate lumen segmentation using L-mode interpolation for three-dimensional intravascular optical coherence tomography. Biomed. Opt. Express.

[66] Pociask E., Malinowski K.P., Ślęzak M., Jaworek-Korjakowska J., Wojakowski W., Roleder T. (2018). Fully automated lumen segmentation method for intracoronary optical coherence tomography. J. Healthc. Eng..

[67] Moraes M.C., Cardenas D.A.C., Furuie S.S. (2013). Automatic lumen segmentation in IVOCT images using binary morphological reconstruction. BioMed. Eng. OnLine.

[68] Joseph S., Adnan A., Adlam D. (2016). Automatic segmentation of coronary morphology using transmittance-based lumen intensity-enhanced intravascular optical coherence tomography images and applying a localized level-set-based active contour method. J. Med. Imaging.

[69] Roy A.G., Conjeti S., Carlier S.G., Dutta P.K., Kastrati A., Laine A.F., Navab N., Katouzian A., Sheet D. (2016). Lumen segmentation in intravascular optical coherence tomography using backscattering tracked and initialized random walks. IEEE J. Biomed. Health Inform..

[70] Essa E., Xie X. (2017). Automatic segmentation of cross-sectional coronary arterial images. Comput. Vis. Image Underst..

[71] Breiman L. (2001). Random forests. Mach. Learn..

[72] Prasad A.M., Iverson L.R., Liaw A. (2006). Newer classification and regression tree techniques: Bagging and random forests for ecological prediction. Ecosystems.

[73] Li K., Wu X., Chen D.Z., Sonka M. (2005). Optimal surface segmentation in volumetric images-a graph-theoretic approach. IEEE Trans. Pattern Anal. Mach. Intell..

[74] Cao Y., Cheng K., Qin X., Yin Q., Li J., Zhu R., Zhao W. (2017). Automatic lumen segmentation in intravascular optical coherence tomography images using level set. Comput. Math. Methods Med..

[75] Macedo M.M.G., Guimarães W.V.N., Galon M.Z., Takimura C.K., Lemos P.A., Gutierrez M.A. (2015). A bifurcation identifier for IV-OCT using orthogonal least squares and supervised machine learning. Comput. Med. Imaging Graph..

[76] Cao Y., Jin Q., Chen Y., Yin Q., Qin X., Li J., Zhu R., Zhao W. (2018). Automatic side branch ostium detection and main vascular segmentation in intravascular optical coherence tomography images. IEEE J. Biomed. Health Inform..

[77] Miyagawa M., Costa M.G.F., Gutierrez M.A., Costa J.P.G.F., Filho C.F.F.C. (2019). Detecting vascular bifurcation in IVOCT images using convolutional neural networks with transfer learning. IEEE Access.

[78] Miyagawa M., Costa M.G.F., Gutierrez M.A., Costa J.P.G.F., Costa Filho C.F. Lumen Segmentation in Optical Coherence Tomography Images Using Convolutional Neural Network. Proceedings of the 2018 40th Annual International Conference of the IEEE Engineering in Medicine and Biology Society (EMBC).

[79] Porto C., Costa Filho C.F., Macedo M.M., Gutierrez M.A., Costa M.G.F. Classification of Bifurcations Regions in IVOCT Images Using Support Vector Machine and Artificial Neural Network Models. Proceedings of the Medical Imaging 2017: Computer-Aided Diagnosis.

[80] Wang A., Eggermont J., Reiber J.H., Dijkstra J. (2014). Fully automated side branch detection in intravascular optical coherence tomography pullback runs. Biomed. Opt. Express.

[81] Quinlan J.R. Bagging, boosting, and C4. 5. Proceedings of the Aaai/iaai.

[82] Freund Y., Schapire R.E. (1997). A decision-theoretic generalization of on-line learning and an application to boosting. J. Comput. Syst. Sci..

[83] Quinlan J.R. (2014). C4. 5: Programs for Machine Learning.

[84] Yang S., Yoon H.-J., Yazdi S.J.M., Lee J.-H. (2020). A novel automated lumen segmentation and classification algorithm for detection of irregular protrusion after stents deployment. Int. J. Med. Robot. Comput. Assist. Surg..

[85] Yong Y.L., Tan L.K., McLaughlin R., Chee K.H., Liew Y.M. (2017). Linear-regression convolutional neural network for fully automated coronary lumen segmentation in intravascular optical coherence tomography. J. Biomed. Opt..

[86] Kingma D.P., Ba J. (2014). Adam: A method for stochastic optimization. arXiv.

[87] Tang J., Lan Y., Chen S., Zhong Y., Huang C., Peng Y., Liu Q., Cheng Y., Chen F., Che W. (2019). Lumen contour segmentation in IVOCT based on N-type CNN. IEEE Access.

[88] Pyxaras S.A., Tu S., Barbato E., Barbati G., Di Serafino L., De Vroey F., Toth G., Mangiacapra F., Sinagra G., De Bruyne B. (2013). Quantitative angiography and optical coherence tomography for the functional assessment of nonobstructive coronary stenoses: Comparison with fractional flow reserve. Am. Heart J..

[89] Westra J., Andersen Birgitte K., Campo G., Matsuo H., Koltowski L., Eftekhari A., Liu T., Di Serafino L., Di Girolamo D., Escaned J. (2018). Diagnostic performance of in-procedure angiography-derived quantitative flow reserve compared to pressure-derived fractional flow feserve: The FAVOR II Europe-Japan study. J. Am. Heart Assoc..

[90] Stone P.H., Saito S., Takahashi S., Makita Y., Nakamura S., Kawasaki T., Takahashi A., Katsuki T., Nakamura S., Namiki A. (2012). Prediction of progression of coronary artery disease and clinical outcomes using vascular profiling of endothelial shear stress and arterial plaque characteristics: The PREDICTION Study. Circulation.

[91] Athanasiou L., Nezami F.R., Galon M.Z., Lopes A.C., Lemos P.A., Hernandez J.M.d.l.T., Ben-Assa E., Edelman E.R. (2018). Optimized computer-aided segmentation and three-dimensional reconstruction using intracoronary optical coherence tomography. IEEE J. Biomed. Health Inform..

[92] Athanasiou L., Bourantas C., Rigas G., Sakellarios A., Exarchos T., Siogkas P., Ricciardi A., Naka K., Papafaklis M., Michalis L. (2014). Methodology for fully automated segmentation and plaque characterization in intracoronary optical coherence tomography images. J. Biomed. Opt..

[93] Balaji A., Kelsey L.J., Majeed K., Schultz C.J., Doyle B.J. (2021). Coronary artery segmentation from intravascular optical coherence tomography using deep capsules. Artif. Intell. Med..

[94] He K., Zhang X., Ren S., Sun J. Identity mappings in deep residual networks. Proceedings of the European Conference on Computer Vision.

[95] LaLonde R., Bagci U. (2018). Capsules for object segmentation. arXiv.

[96] Sabour S., Frosst N., Hinton G.E. (2017). Dynamic routing between capsules. arXiv.

[97] He K., Zhang X., Ren S., Sun J. Deep residual learning for image recognition. Proceedings of the IEEE Conference on Computer Vision and Pattern Recognition.

[98] Long J., Shelhamer E., Darrell T. Fully convolutional networks for semantic segmentation. Proceedings of the IEEE Conference on Computer Vision and Pattern Recognition.

[99] Chen L.-C., Papandreou G., Schroff F., Adam H. (2017). Rethinking atrous convolution for semantic image segmentation. arXiv.

[100] Zahnd G., Hoogendoorn A., Combaret N., Karanasos A., Péry E., Sarry L., Motreff P., Niessen W., Regar E., van Soest G. (2017). Contour segmentation of the intima, media, and adventitia layers in intracoronary OCT images: Application to fully automatic detection of healthy wall regions. Int. J. Comput. Assist. Radiol. Surg..

[101] Abdolmanafi A., Duong L., Dahdah N., Cheriet F. (2017). Deep feature learning for automatic tissue classification of coronary artery using optical coherence tomography. Biomed. Opt. Express.

[102] Chen Z., Pazdernik M., Zhang H., Wahle A., Guo Z., Bedanova H., Kautzner J., Melenovsky V., Kovarnik T., Sonka M. (2018). Quantitative 3D analysis of coronary wall morphology in heart transplant patients: OCT-assessed cardiac allograft vasculopathy progression. Med. Image Anal..

[103] Pazdernik M., Chen Z., Bedanova H., Kautzner J., Melenovsky V., Karmazin V., Malek I., Tomasek A., Ozabalova E., Krejci J. (2018). Early detection of cardiac allograft vasculopathy using highly automated 3-dimensional optical coherence tomography analysis. J. Heart Lung Transplant..

[104] Yin Y., Zhang X., Williams R., Wu X., Anderson D.D., Sonka M. (2010). LOGISMOS—Layered optimal graph image segmentation of multiple objects and surfaces: Cartilage segmentation in the knee joint. IEEE Trans. Med. Imaging.

[105] Zhang H., Lee K., Chen Z., Kashyap S., Sonka M., Zhou S.K., Rueckert D., Fichtinger G. (2020). Chapter 11—LOGISMOS-JEI: Segmentation Using Optimal Graph Search and Just-Enough Interaction. Handbook of Medical Image Computing and Computer Assisted Intervention.

[106] Jia Y., Shelhamer E., Donahue J., Karayev S., Long J., Girshick R., Guadarrama S., Darrell T. Caffe: Convolutional architecture for fast feature embedding. Proceedings of the 22nd ACM International Conference on Multimedia.

[107] Otsuka K., Villiger M., Nadkarni S.K., Bouma B.E. (2019). Intravascular polarimetry for tissue characterization of coronary atherosclerosis. Circ. Rep..

[108] Otsuka K., Villiger M., Nadkarni S.K., Bouma B.E. (2020). Intravascular polarimetry: Clinical translation and future applications of catheter-based polarization sensitive optical frequency domain imaging. Front. Cardiovasc. Med..

[109] Villiger M., Otsuka K., Karanasos A., Doradla P., Ren J., Lippok N., Shishkov M., Daemen J., Diletti R., van Geuns R.-J. (2018). Coronary plaque microstructure and composition modify optical polarization: A new endogenous contrast mechanism for optical frequency domain imaging. JACC Cardiovasc. Imaging.

[110] Haft-Javaherian M., Villiger M., Otsuka K., Daemen J., Libby P., Golland P., Bouma B.E. (2021). Segmentation of anatomical layers and artifacts in intravascular polarization sensitive optical coherence tomography using attending physician and boundary cardinality lost terms. arXiv.

[111] Xu B., Wang N., Chen T., Li M. (2015). Empirical evaluation of rectified activations in convolutional network. arXiv.

[112] Li J., Montarello N.J., Hoogendoorn A., Verjans J.W., Bursill C.A., Peter K., Nicholls S.J., McLaughlin R.A., Psaltis P.J. (2021). Multimodality intravascular imaging of high-risk coronary plaque. JACC Cardiovasc. Imaging.

[113] Olender M.L., Athanasiou L.S., Hernández J.M.d.l.T., Ben-Assa E., Nezami F.R., Edelman E.R. (2019). A Mechanical Approach for Smooth Surface Fitting to Delineate Vessel Walls in Optical Coherence Tomography Images. IEEE Trans. Med. Imaging.

[114] Olender M.L., Athanasiou L.S., José M., Camarero T.G., Cascón J.D., Consuegra-Sanchez L., Edelman E.R. Estimating the internal elastic membrane cross-sectional area of coronary arteries autonomously using optical coherence tomography images. Proceedings of the 2017 IEEE EMBS International Conference on Biomedical & Health Informatics (BHI).

[115] Prabhu D.S., Bezerra H.G., Kolluru C., Gharaibeh Y., Mehanna E., Wu H., Wilson D.L. (2019). Automated A-line coronary plaque classification of intravascular optical coherence tomography images using handcrafted features and large datasets. J. Biomed. Opt..

[116] Peng H., Long F., Ding C. (2005). Feature selection based on mutual information criteria of max-dependency, max-relevance, and min-redundancy. IEEE Trans. Pattern Anal. Mach. Intell..

[117] Parmar C., Grossmann P., Bussink J., Lambin P., Aerts H.J. (2015). Machine learning methods for quantitative radiomic biomarkers. Sci. Rep..

[118] Krähenbühl P., Koltun V. (2011). Efficient inference in fully connected crfs with gaussian edge potentials. Adv. Neural Inf. Process. Syst..

[119] Leung T., Malik J. (2001). Representing and recognizing the visual appearance of materials using three-dimensional textons. Int. J. Comput. Vis..

[120] Zhang C., Guo X., Guo X., Molony D., Li H., Samady H., Giddens D.P., Athanasiou L., Tang D., Nie R. (2020). Machine learning model comparison for automatic segmentation of intracoronary optical coherence tomography and plaque cap thickness quantification. Comput. Model. Eng. Sci..

[121] Huang G., Liu Z., Van Der Maaten L., Weinberger K.Q. Densely connected convolutional networks. Proceedings of the IEEE Conference on Computer Vision and Pattern Recognition.

[122] Jégou S., Drozdzal M., Vazquez D., Romero A., Bengio Y. The one hundred layers tiramisu: Fully convolutional densenets for semantic segmentation. Proceedings of the IEEE Conference on Computer Vision and Pattern Recognition Workshops.

[123] Lv R., Maehara A., Matsumura M., Wang L., Wang Q., Zhang C., Guo X., Samady H., Giddens D.P., Zheng J. (2020). Using optical coherence tomography and intravascular ultrasound imaging to quantify coronary plaque cap thickness and vulnerability: A pilot study. BioMed. Eng. OnLine.

[124] Zahnd G., Karanasos A., van Soest G., Regar E., Niessen W., Gijsen F., van Walsum T. (2015). Quantification of fibrous cap thickness in intracoronary optical coherence tomography with a contour segmentation method based on dynamic programming. Int. J. Comput. Assist. Radiol. Surg..

[125] Wang Z., Chamie D., Bezerra H.G., Yamamoto H., Kanovsky J., Wilson D.L., Costa M.A., Rollins A.M. (2012). Volumetric quantification of fibrous caps using intravascular optical coherence tomography. Biomed. Opt. Express.

[126] Zhang C., Li H., Guo X., Molony D., Guo X., Samady H., Giddens D.P., Athanasiou L., Nie R., Cao J. (2019). Convolution neural networks and support vector machines for automatic segmentation of intracoronary optical coherence tomography. Mol. Cell. Biomech..

[127] Yang J., Zhang B., Wang H., Lin F., Han Y., Liu X. (2019). Automated characterization and classification of coronary atherosclerotic plaques for intravascular optical coherence tomography. Biocybern. Biomed. Eng..

[128] Wang P., Zhu H., Ling X. (2020). Intravascular optical coherence tomography image segmentation based on Gaussian mixture model and adaptive fourth-order PDE. Signal Image Video Process..

[129] Rico-Jimenez J.J., Campos-Delgado D.U., Villiger M., Otsuka K., Bouma B.E., Jo J.A. (2016). Automatic classification of atherosclerotic plaques imaged with intravascular OCT. Biomed. Opt. Express.

[130] Gessert N., Lutz M., Heyder M., Latus S., Leistner D.M., Abdelwahed Y.S., Schlaefer A. (2019). Automatic plaque detection in IVOCT pullbacks using convolutional neural networks. IEEE Trans. Med. Imaging.

[131] Athanasiou L.S., Olender M.L., José M., Ben-Assa E., Edelman E.R. A deep learning approach to classify atherosclerosis using intracoronary optical coherence tomography. Proceedings of the Medical Imaging 2019: Computer-Aided Diagnosis.

[132] Abdolmanafi A., Duong L., Dahdah N., Adib I.R., Cheriet F. (2018). Characterization of coronary artery pathological formations from OCT imaging using deep learning. Biomed. Opt. Express.

[133] Abdolmanafi A., Cheriet F., Duong L., Ibrahim R., Dahdah N. (2019). An automatic diagnostic system of coronary artery lesions in Kawasaki disease using intravascular optical coherence tomography imaging. J. Biophotonics.

[134] Abdolmanafi A., Duong L., Ibrahim R., Dahdah N. (2021). A deep learning-based model for characterization of atherosclerotic plaque in coronary arteries using optical coherence tomography images. Med. Phys..

[135] Li L., Jia T. (2019). Optical coherence tomography vulnerable plaque segmentation based on deep residual U-net. Rev. Cardiovasc. Med..

[136] Huang Y., He C., Wang J., Miao Y., Zhu T., Zhou P., Li Z. (2018). Intravascular optical coherence tomography image segmentation based on support vector machine algorithm. MCB Mol. Cell. Biomech..

[137] You Y.-L., Kaveh M. (2000). Fourth-order partial differential equations for noise removal. IEEE Trans. Image Process..

[138] Nguyen T.M., Wu Q.J. (2012). Fast and robust spatially constrained Gaussian mixture model for image segmentation. IEEE Trans. Circuits Syst. Video Technol..

[139] Kumar R., Srivastava S., Srivastava R. (2017). A fourth order PDE based fuzzy c-means approach for segmentation of microscopic biopsy images in presence of Poisson noise for cancer detection. Comput. Methods Programs Biomed..

[140] Trivedi M.M., Bezdek J.C. (1986). Low-level segmentation of aerial images with fuzzy clustering. IEEE Trans. Syst. Man Cybern..

[141] Sfikas G., Nikou C., Galatsanos N. Robust image segmentation with mixtures of Student’s t-distributions. Proceedings of the 2007 IEEE International Conference on Image Processing.

[142] Titterington D.M., Afm S., Smith A.F., Makov U. (1985). Statistical Analysis of Finite Mixture Distributions.

[143] Bi H., Tang H., Yang G., Shu H., Dillenseger J.-L. (2018). Accurate image segmentation using Gaussian mixture model with saliency map. Pattern Anal. Appl..

[144] Liu X., Du J., Yang J., Xiong P., Liu J., Lin F. (2020). Coronary artery fibrous plaque detection based on multi-scale convolutional neural networks. J. Signal Process. Syst..

[145] Simonyan K., Zisserman A. (2014). Very deep convolutional networks for large-scale image recognition. arXiv.

[146] Liu W., Anguelov D., Erhan D., Szegedy C., Reed S., Fu C.-Y., Berg A.C. SSD: Single shot multibox detector. Proceedings of the European Conference on Computer Vision.

[147] Redmon J., Divvala S., Girshick R., Farhadi A. You only look once: Unified, real-time object detection. Proceedings of the IEEE Conference on Computer Vision and Pattern Recognition.

[148] Redmon J., Farhadi A. YOLO9000: Better, faster, stronger. Proceedings of the IEEE Conference on Computer Vision and Pattern Recognition.

[149] Redmon J., Farhadi A. (2018). Yolov3: An incremental improvement. arXiv.

[150] Liu R., Zhang Y., Zheng Y., Liu Y., Zhao Y., Yi L. (2019). Automated detection of vulnerable plaque for intravascular optical coherence tomography images. Cardiovasc. Eng. Technol..

[151] Gerbaud E., Weisz G., Tanaka A., Luu R., Osman H.A.S.H., Baldwin G., Coste P., Cognet L., Waxman S., Zheng H. (2020). Plaque burden can be assessed using intravascular optical coherence tomography and a dedicated automated processing algorithm: A comparison study with intravascular ultrasound. Eur. Heart J. Cardiovasc. Imaging.

[152] Isidori F., Lella E., Marco V., Albertucci M., Ozaki Y., La Manna A., Biccirè F.G., Romagnoli E., Bourantas C.V., Paoletti G. (2021). Adoption of a new automated optical coherence tomography software to obtain a lipid plaque spread-out plot. Int. J. Cardiovasc. Imaging.

[153] Rico-Jimenez J.J., Campos-Delgado D.U., Buja L.M., Vela D., Jo J.A. (2019). Intravascular optical coherence tomography method for automated detection of macrophage infiltration within atherosclerotic coronary plaques. Atherosclerosis.

[154] Shibutani H., Fujii K., Ueda D., Kawakami R., Imanaka T., Kawai K., Matsumura K., Hashimoto K., Yamamoto A., Hao H. (2021). Automated classification of coronary atherosclerotic plaque in optical frequency domain imaging based on deep learning. Atherosclerosis.

[155] Kolluru C., Prabhu D., Gharaibeh Y., Bezerra H., Guagliumi G., Wilson D. (2018). Deep neural networks for A-line-based plaque classification in coronary intravascular optical coherence tomography images. J. Med. Imaging.

[156] Lee J., Prabhu D., Kolluru C., Gharaibeh Y., Zimin V.N., Dallan L.A.P., Bezerra H.G., Wilson D.L. (2020). Fully automated plaque characterization in intravascular OCT images using hybrid convolutional and lumen morphology features. Sci. Rep..

[157] Lee J., Prabhu D., Kolluru C., Gharaibeh Y., Zimin V.N., Bezerra H.G., Wilson D.L. (2019). Automated plaque characterization using deep learning on coronary intravascular optical coherence tomographic images. Biomed. Opt. Express.

[158] Badrinarayanan V., Kendall A., Cipolla R. (2017). Segnet: A deep convolutional encoder-decoder architecture for image segmentation. IEEE Trans. Pattern Anal. Mach. Intell..

[159] Chen L.-C., Zhu Y., Papandreou G., Schroff F., Adam H. Encoder-decoder with atrous separable convolution for semantic image segmentation. Proceedings of the European Conference on Computer Vision (ECCV).

[160] Russakovsky O., Deng J., Su H., Krause J., Satheesh S., Ma S., Huang Z., Karpathy A., Khosla A., Bernstein M. (2015). Imagenet large scale visual recognition challenge. Int. J. Comput. Vis..

[161] Cheimariotis G.-A., Riga M., Haris K., Toutouzas K., Katsaggelos A.K., Maglaveras N. (2021). Automatic classification of A-lines in intravascular OCT images using deep learning and estimation of attenuation coefficients. Appl. Sci..

[162] Liu S., Sotomi Y., Eggermont J., Nakazawa G., Torii S., Ijichi T., Onuma Y., Serruys P.W., Lelieveldt B.P., Dijkstra J. (2017). Tissue characterization with depth-resolved attenuation coefficient and backscatter term in intravascular optical coherence tomography images. J. Biomed. Opt..

[163] Ughi G.J., Adriaenssens T., Sinnaeve P., Desmet W., D’hooge J. (2013). Automated tissue characterization of in vivo atherosclerotic plaques by intravascular optical coherence tomography images. Biomed. Opt. Express.

[164] Krizhevsky A., Sutskever I., Hinton G.E. (2012). Imagenet classification with deep convolutional neural networks. Adv. Neural Inf. Process. Syst..

[165] Szegedy C., Vanhoucke V., Ioffe S., Shlens J., Wojna Z. Rethinking the inception architecture for computer vision. Proceedings of the IEEE Conference on Computer Vision and Pattern Recognition.

[166] Zhang Z. Improved adam optimizer for deep neural networks. Proceedings of the 2018 IEEE/ACM 26th International Symposium on Quality of Service (IWQoS).

[167] He C., Wang J., Yin Y., Li Z. (2020). Automated classification of coronary plaque calcification in OCT pullbacks with 3D deep neural networks. J. Biomed. Opt..

[168] Avital Y., Madar A., Arnon S., Koifman E. (2021). Identification of coronary calcifications in optical coherence tomography imaging using deep learning. Sci. Rep..

[169] Lee J., Gharaibeh Y., Kolluru C., Zimin V.N., Dallan L.A.P., Kim J.N., Bezerra H.G., Wilson D.L. (2020). Segmentation of coronary calcified plaque in intravascular OCT images using a two-step deep learning approach. IEEE Access.

[170] Gharaibeh Y., Prabhu D., Kolluru C., Lee J., Zimin V., Bezerra H., Wilson D. (2019). Coronary calcification segmentation in intravascular OCT images using deep learning: Application to calcification scoring. J. Med. Imaging.

[171] Cordts M., Omran M., Ramos S., Rehfeld T., Enzweiler M., Benenson R., Franke U., Roth S., Schiele B. The cityscapes dataset for semantic urban scene understanding. Proceedings of the IEEE Conference on Computer Vision and Pattern Recognition.

[172] Kolluru C., Lee J., Gharaibeh Y., Bezerra H.G., Wilson D.L. (2021). Learning with fewer images via image clustering: Application to intravascular OCT image segmentation. IEEE Access.

[173] Shlofmitz E., Iantorno M., Waksman R. (2019). Restenosis of drug-eluting stents. Circ. Cardiovasc. Interv..

[174] Nam H.S., Kim C.-S., Lee J.J., Song J.W., Kim J.W., Yoo H. (2016). Automated detection of vessel lumen and stent struts in intravascular optical coherence tomography to evaluate stent apposition and neointimal coverage. Med. Phys..

[175] Wu P., Gutiérrez-Chico J.L., Tauzin H., Yang W., Li Y., Yu W., Chu M., Guillon B., Bai J., Meneveau N. (2020). Automatic stent reconstruction in optical coherence tomography based on a deep convolutional model. Biomed. Opt. Express.

[176] Cao Y., Jin Q., Lu Y., Jing J., Chen Y., Yin Q., Qin X., Li J., Zhu R., Zhao W. (2018). Automatic analysis of bioresorbable vascular scaffolds in intravascular optical coherence tomography images. Biomed. Opt. Express.

[177] Zysk A.M., Nguyen F.T., Oldenburg A.L., Marks D.L., Boppart S.A. (2007). Optical coherence tomography: A review of clinical development from bench to bedside. J. Biomed. Opt..

[178] Jiang X., Zeng Y., Xiao S., He S., Ye C., Qi Y., Zhao J., Wei D., Hu M., Chen F. (2020). Automatic detection of coronary metallic stent struts based on YOLOv3 and R-FCN. Comput. Math. Methods Med..

[179] Amrute J.M., Athanasiou L.S., Rikhtegar F., de la Torre Hernández J.M., Camarero T.G., Edelman E.R. (2018). Polymeric endovascular strut and lumen detection algorithm for intracoronary optical coherence tomography images. J. Biomed. Opt..

[180] Lau Y.S., Tan L.K., Chan C.K., Chee K.H., Liew Y.M. (2021). Automated segmentation of metal stent and bioresorbable vascular scaffold in intravascular optical coherence tomography images using deep learning architectures. Phys. Med. Biol..

[181] Sandler M., Howard A., Zhu M., Zhmoginov A., Chen L.-C. Mobilenetv2: Inverted residuals and linear bottlenecks. Proceedings of the IEEE Conference on Computer Vision and Pattern Recognition.

[182] Lu H., Lee J., Ray S., Tanaka K., Bezerra H.G., Rollins A.M., Wilson D.L. (2019). Automated stent coverage analysis in intravascular OCT (IVOCT) image volumes using a support vector machine and mesh growing. Biomed. Opt. Express.

[183] Chang C.-C., Lin C.-J. (2011). LIBSVM: A library for support vector machines. ACM Trans. Intell. Syst. Technol..

[184] Lu H., Lee J., Jakl M., Wang Z., Cervinka P., Bezerra H.G., Wilson D.L. (2020). Application and evaluation of highly automated software for comprehensive stent analysis in intravascular optical coherence tomography. Sci. Rep..

[185] Lin G., Milan A., Shen C., Reid I. Refinenet: Multi-path refinement networks for high-resolution semantic segmentation. Proceedings of the IEEE Conference on Computer Vision and Pattern Recognition.

[186] O’Brien C.C., Kolandaivelu K., Brown J., Lopes A.C., Kunio M., Kolachalama V.B., Edelman E.R. (2016). Constraining OCT with Knowledge of Device Design Enables High Accuracy Hemodynamic Assessment of Endovascular Implants. PLoS ONE.

[187] Chiastra C., Montin E., Bologna M., Migliori S., Aurigemma C., Burzotta F., Celi S., Dubini G., Migliavacca F., Mainardi L. (2017). Reconstruction of stented coronary arteries from optical coherence tomography images: Feasibility, validation, and repeatability of a segmentation method. PLoS ONE.

[188] Wang A., Eggermont J., Dekker N., Garcia-Garcia H.M., Pawar R., Reiber J.H., Dijkstra J. (2013). Automatic stent strut detection in intravascular optical coherence tomographic pullback runs. Int. J. Cardiovasc. Imaging.

[189] Migliori S., Chiastra C., Bologna M., Montin E., Dubini G., Aurigemma C., Fedele R., Burzotta F., Mainardi L., Migliavacca F. (2017). A framework for computational fluid dynamic analyses of patient-specific stented coronary arteries from optical coherence tomography images. Med. Eng. Phys..

[190] Elliott M.R., Kim D., Molony D.S., Morris L., Samady H., Joshi S., Timmins L.H. (2019). Establishment of an automated algorithm utilizing optical coherence tomography and micro-computed tomography imaging to reconstruct the 3-D deformed stent geometry. IEEE Trans. Med. Imaging.

[191] Tsantis S., Kagadis G.C., Katsanos K., Karnabatidis D., Bourantas G., Nikiforidis G.C. (2012). Automatic vessel lumen segmentation and stent strut detection in intravascular optical coherence tomography. Med. Phys..

[192] Ughi G.J., Adriaenssens T., Onsea K., Kayaert P., Dubois C., Sinnaeve P., Coosemans M., Desmet W., D’hooge J. (2012). Automatic segmentation of in-vivo intra-coronary optical coherence tomography images to assess stent strut apposition and coverage. Int. J. Cardiovasc. Imaging.

[193] Li J., Li X., Mohar D., Raney A., Jing J., Zhang J., Johnston A., Liang S., Ma T., Shung K.K. (2014). Integrated IVUS-OCT for real-time imaging of coronary atherosclerosis. JACC Cardiovasc. Imaging.

[194] Cha S.-H. (2007). Comprehensive survey on distance/similarity measures between probability density functions. City.

[195] Yeghiazaryan V., Voiculescu I. (2018). Family of boundary overlap metrics for the evaluation of medical image segmentation. J. Med. Imaging.

[196] Baxter J.S.H., Jannin P. (2022). Bias in machine learning for computer-assisted surgery and medical image processing. Comput. Assist. Surg..

[197] Sengupta Partho P., Shrestha S., Berthon B., Messas E., Donal E., Tison Geoffrey H., Min James K., D’hooge J., Voigt J.-U., Dudley J. (2020). Proposed requirements for cardiovascular imaging-related machine learning evaluation (PRIME): A checklist. JACC Cardiovasc. Imaging.

[198] Cao Y., Fang Z., Wu Y., Zhou D.-X., Gu Q. (2019). Towards understanding the spectral bias of deep learning. arXiv.

[199] Statnikov A., Wang L., Aliferis C.F. (2008). A comprehensive comparison of random forests and support vector machines for microarray-based cancer classification. BMC Bioinform..

[200] Vokinger K.N., Feuerriegel S., Kesselheim A.S. (2021). Mitigating bias in machine learning for medicine. Commun. Med..

[201] Bernard O., Lalande A., Zotti C., Cervenansky F., Yang X., Heng P.A., Cetin I., Lekadir K., Camara O., Ballester M.A.G. (2018). Deep learning techniques for automatic MRI cardiac multi-structures segmentation and diagnosis: Is the problem solved?. IEEE Trans. Med. Imaging.

[202] MONAI Medical Open Network for Artificial Intelligence. https://monai.io/index.html.

[203] Yi X., Walia E., Babyn P. (2019). Generative adversarial network in medical imaging: A review. Med. Image Anal..

[204] Kazeminia S., Baur C., Kuijper A., van Ginneken B., Navab N., Albarqouni S., Mukhopadhyay A. (2020). GANs for medical image analysis. Artif. Intell. Med..

[205] Chen M., Shi X., Zhang Y., Wu D., Guizani M. (2021). Deep feature learning for medical image analysis with convolutional autoencoder neural network. IEEE Trans. Big Data.

[206] Kadry K., Olender M.L., Marlevi D., Edelman E.R., Nezami F.R. (2021). A platform for high-fidelity patient-specific structural modelling of atherosclerotic arteries: From intravascular imaging to three-dimensional stress distributions. J. R. Soc. Interface.

[207] Griese F., Latus S., Schlüter M., Graeser M., Lutz M., Schlaefer A., Knopp T. (2020). In-Vitro MPI-guided IVOCT catheter tracking in real time for motion artifact compensation. PLoS ONE.

[208] Wu W., Samant S., de Zwart G., Zhao S., Khan B., Ahmad M., Bologna M., Watanabe Y., Murasato Y., Burzotta F. (2020). 3D reconstruction of coronary artery bifurcations from coronary angiography and optical coherence tomography: Feasibility, validation, and reproducibility. Sci. Rep..

[209] Zhu Y., Zhu F., Ding Z., Tao K., Lai T., Kuang H., Hua P., Shang M., Hu J., Yu Y. (2021). Three-dimensional spatial reconstruction of coronary arteries based on fusion of intravascular optical coherence tomography and coronary angiography. J. Biophotonics.

[210] Wang J., Paritala P.K., Mendieta J.B., Komori Y., Raffel O.C., Gu Y., Li Z. (2020). Optical coherence tomography-based patient-specific coronary artery reconstruction and fluid–structure interaction simulation. Biomech. Model. Mechanobiol..

[211] Schaap M., Metz C.T., van Walsum T., van der Giessen A.G., Weustink A.C., Mollet N.R., Bauer C., Bogunović H., Castro C., Deng X. (2009). Standardized evaluation methodology and reference database for evaluating coronary artery centerline extraction algorithms. Med. Image Anal..

[212] Hajhosseiny R., Munoz C., Cruz G., Khamis R., Kim W.Y., Prieto C., Botnar R.M. (2021). Coronary magnetic resonance angiography in chronic coronary syndromes. Front. Cardiovasc. Med..

[213] Sakuma H. (2011). Coronary CT versus MR angiography: The role of MR angiography. Radiology.

[214] Li J., Ma T., Zhou Q., Chen Z., Zhou Q., Chen Z. (2020). The integration of IVUS and OCT. Multimodality Imaging: For Intravascular Application.

[215] Fujii K., Hao H., Shibuya M., Imanaka T., Fukunaga M., Miki K., Tamaru H., Sawada H., Naito Y., Ohyanagi M. (2015). Accuracy of OCT, grayscale IVUS, and their combination for the diagnosis of coronary TCFA. JACC Cardiovasc. Imaging.

[216] Fracassi F., Crea F., Sugiyama T., Yamamoto E., Uemura S., Vergallo R., Porto I., Lee H., Fujimoto J., Fuster V. (2019). Healed culprit plaques in patients with acute coronary syndromes. J. Am. Coll. Cardiol..

[217] Nadkarni S.K., Pierce M.C., Park B.H., de Boer J.F., Whittaker P., Bouma B.E., Bressner J.E., Halpern E., Houser S.L., Tearney G.J. (2007). Measurement of collagen and smooth muscle cell content in atherosclerotic plaques using polarization-sensitive optical coherence tomography. J. Am. Coll. Cardiol..

[218] MacRitchie N., Grassia G., Noonan J., Garside P., Graham D., Maffia P. (2018). Molecular imaging of atherosclerosis: Spotlight on Raman spectroscopy and surface-enhanced Raman scattering. Heart.

[219] Osborn E.A., Jaffer F.A. (2013). The advancing clinical impact of molecular imaging in CVD. JACC Cardiovasc. Imaging.

[220] Tarkin J.M., Joshi F.R., Rudd J.H. (2014). PET imaging of inflammation in atherosclerosis. Nat. Rev. Cardiol..

[221] Ughi G.J., Wang H., Gerbaud E., Gardecki J.A., Fard A.M., Hamidi E., Vacas-Jacques P., Rosenberg M., Jaffer F.A., Tearney G.J. (2016). Clinical characterization of coronary atherosclerosis with dual-modality OCT and near-infrared autofluorescence imaging. JACC Cardiovasc. Imaging.

[222] Ali Z.A., Karimi Galougahi K., Maehara A., Shlofmitz R.A., Ben-Yehuda O., Mintz G.S., Stone G.W. (2017). Intracoronary optical coherence tomography 2018: Current status and future directions. JACC Cardiovasc. Interv..

[223] Calvert Patrick A., Obaid Daniel R., O’Sullivan M., Shapiro Leonard M., McNab D., Densem Cameron G., Schofield Peter M., Braganza D., Clarke Sarah C., Ray Kausik K. (2011). Association between IVUS findings and adverse outcomes in patients with coronary artery disease. JACC Cardiovasc. Imaging.

[224] Baruah V., Zahedivash A., Hoyt T., McElroy A., Vela D., Buja L.M., Cabe A., Oglesby M., Estrada A., Antonik P. (2020). Automated coronary plaque characterization with intravascular optical coherence tomography and smart-algorithm approach. JACC Cardiovasc. Imaging.

[225] Holzapfel G.A. (2001). Biomechanics of soft tissue. Handb. Mater. Behav. Models.

[226] Hollander Y., Durban D., Lu X., Kassab G.S., Lanir Y. (2011). Constitutive modeling of coronary arterial media—Comparison of three model classes. J. Biomech. Eng..

[227] Holzapfel G.A., Gasser T.C., Ogden R.W. (2000). A new constitutive framework for arterial wall mechanics and a comparative study of material models. J. Elast. Phys. Sci. Solids.

[228] Holzapfel G.A., Gasser T.C., Stadler M. (2002). A structural model for the viscoelastic behavior of arterial walls: Continuum formulation and finite element analysis. Eur. J. Mech. A/Solids.

[229] Khaniki H.B., Ghayesh M.H., Chin R., Amabili M. (2021). Large amplitude vibrations of imperfect porous-hyperelastic beams via a modified strain energy. J. Sound Vib..

[230] Rivlin R.S. (1948). Large elastic deformations of isotropic materials IV. Further developments of the general theory. Philos. Trans. R. Soc. Lond. Ser. A Math. Phys. Sci..

[231] Narayanan B., Olender M.L., Marlevi D., Edelman E.R., Nezami F.R. (2021). An inverse method for mechanical characterization of heterogeneous diseased arteries using intravascular imaging. Sci. Rep..

[232] Baldewsing R.A., Schaar J.A., Mastik F., Oomens C.W., van der Steen A.F. (2005). Assessment of vulnerable plaque composition by matching the deformation of a parametric plaque model to measured plaque deformation. IEEE Trans. Med. Imaging.

[233] Baldewsing R.A., Danilouchkine M.G., Mastik F., Schaar J.A., Serruys P.W., van der Steen A.F. (2008). An inverse method for imaging the local elasticity of atherosclerotic coronary plaques. IEEE Trans. Inf. Technol. Biomed..

[234] Le Floc’h S., Ohayon J., Tracqui P., Finet G., Gharib A.M., Maurice R.L., Cloutier G., Pettigrew R.I. (2009). Vulnerable atherosclerotic plaque elasticity reconstruction based on a segmentation-driven optimization procedure using strain measurements: Theoretical framework. IEEE Trans. Med. Imaging.

[235] Taylor J., Fenner J. (2019). The challenge of clinical adoption—The insurmountable obstacle that will stop machine learning?. BJR|Open.

[236] Bhattacharyya A. (1943). On a measure of divergence between two statistical populations defined by their probability distributions. Bull. Calcutta Math. Soc..

[237] Cohen J. (1960). A coefficient of agreement for nominal scales. Educ. Psychol. Meas..

[238] Lawrence I., Lin K. (1989). A concordance correlation coefficient to evaluate reproducibility. Biometrics.

[239] Dice L.R. (1945). Measures of the amount of ecologic association between species. Ecology.

[240] Jaccard P. (1912). The distribution of the flora in the alpine zone. 1. New Phytol..

[241] Kullback S., Leibler R.A. (1951). On information and sufficiency. Ann. Math. Stat..

